# Enhancing flexural behavior and ductility of fixed-end RC beams using high-performance fiber-reinforced cementitious composites

**DOI:** 10.1038/s41598-025-25190-2

**Published:** 2025-11-21

**Authors:** Mohammad Kazem Sharbatdar, Amir Ghods, Khaled Sennah

**Affiliations:** 1 Faculty of Civil Engineering, Semnan University, Semnan, Iran; 2 Civil Engineering Department, Toronto Metropolitan University, Toronto, ON Canada

**Keywords:** Two fixed-end, High performance, Reinforced concrete, Steel fibers, Ductility, Engineering, Materials science

## Abstract

This paper investigates the effect of High-Performance Fiber-Reinforced Cementitious Composite (HPFRCC) with steel fibers on two fixed-end beams. Eight beams with identical sections were tested: two reference specimens and six reinforced with 1% or 2% steel fibers, with varied stirrup spacing near the beam ends. Results showed that fiber addition and stirrup adjustments markedly improved ductility and energy absorption. With 1% fibers, ductility nearly doubled, while 2% fibers more than doubled load capacity and tripled energy ductility. Adjusting loading points further enhanced strength and ductility. Overall, HPFRCC beams absorbed about 12% more energy than conventional concrete and extended the plastic hinge zone by nearly 50%.

## Introduction

In the past few years, concrete technology has advanced significantly, giving rise to the creation of high-performance cement (HPCs). These HPCs stand out as a unique type of composite cement where individual fibers are integrated into conventional concrete. Among these highly efficient concrete innovations, one that has gained prominence is High-Performance Concrete (HPC). In 2013, Naaman and Reinhardt introduced materials exhibiting strain-hardening characteristics in the tensile phase of the stress–strain curve, coining the term High-Performance Fiber-Reinforced Cementitious Composite (HPFRCC)^1^. Its strain-hardening behavior under tensile loading characterizes HPFRCC after initial cracking, accompanied by the formation of multiple cracks until significant strains are reached. In 2003, Chanvillard and Riguad reported an experimental study on HPFRCC characteristics^2^. This marked an improvement from earlier studies by Fischer and others, where tensile strengths ranged from 4 to 6 MPa and tensile stress levels were between 3 to 5 percent^3^. Recognizing these materials’ strain-hardening properties, the US RILEM Steering Committee emphasized this characteristic, leading to the term "Strain Hardening Cement Composites" (SHCC)^4^. Additionally, a notable HPFRCC variant known as CARDIFRC, developed by researchers Farhat et al. (2006) at Cardiff University, incorporated concrete with a remarkable compressive strength of 200 MPa and a tensile strength of 27 MPa^5^. UHPFRC exhibited impressive mechanical properties, with tensile strengths exceeding 10 MPa, compressive strengths surpassing 150 MPa, and ultimate strains of at least 0.005^6^. Researchers also conducted experiments under uniaxial, biaxial, and triaxial compressive loads to investigate the strain-hardening behavior intrinsic to HPFRCC materials^6^.

The experimental findings revealed that employing shorter fibers can substantially enhance strength and ductility, particularly in situations involving uniaxial and biaxial loading. Furthermore, the results suggested that in triaxial compressive tests characterized by high external confining pressure, the confining effect attributable to reinforcement fibers is negligible, as observed in the study conducted by Sirijaroonchai^7^. Guler et al.^8,9^ conducted material and structural studies on the use of hybrid steel fibers at elevated temperatures and on concrete-filled square stub columns subjected to axial compression. The ultimate deformation properties of HPFRCC beams may be affected by compressive strength, loading type, and the ratio of tensile reinforcement^10^. Their findings demonstrated that transitioning from a concentrated load at the midpoint of the span to a uniform load can significantly enhance the plastic hinge rotation capacity. In a related study, Hemmati et al. employed numerical techniques through the utilization of HPFRCC materials. A comprehensive comparison was made between the original concrete frame and the HPFRCC frame. The outcomes of this analysis indicated that the utilization of HPFRCC materials leads to a notable improvement in both the load-carrying capacity and ductility of these frames^11^. Researchers in recent years have delved into several significant aspects of HPFRCC and its applications. Investigating the growth and development of cracks within HPFRCC materials was a crucial concern addressed by Ghasemi et al.^12^. Analyzing the optimization and flexural performance of HPFRCC, as explored by Meng and Khayat, and further researched by Meng et al.^13,14^. Examining the tensile strain-hardening behavior of HPFRCC, a pivotal aspect investigated by Li et al.^15^. It has been determined that moment redistribution, up to a maximum limit of 25%, does not induce significant alterations in cracking behavior and beam curvature. This level of bending moment aligns with the predictions of elastic theory. Scholz examined the impact of slender and rigid beams on moment redistribution in continuous beams, employing the concept of ductility^16^. He subsequently compared his proposed approach with the allowable moment redistribution specified in the Canadian Code (CSA-A23.3-04). In a separate study, Lin and Chin employed 26 reinforced concrete beams to investigate the influence of longitudinal and transverse reinforcement as well as the compressive strength of the concrete on ductility and moment redistribution^17^. Their findings revealed that transverse steel bars can effectively confine the concrete, leading to increased moment redistribution. Moreover, reducing the amount of tensile steel and elevating the compressive strength of the concrete were found to enhance both ductility and moment redistribution.

Mishra and Li and Naaman et al. compared crack patterns in reinforced concrete (RC) beams and composite RC beams with an underlying HPFRCC layer through point load tests^18^. Hemmati et al. (2014) examined the impact of HPFRCC layer thickness in high-performance concrete beams during two-point flexural testing. Their study revealed that HPFRCC-constructed beams displayed superior ductility compared to those made with normal concrete^19^. In recent decades, researchers have explored several tests on this material^20^. Furthermore, moment redistribution has been a subject of extensive investigation in recent decades, with researchers employing both theoretical and experimental approaches to explore this critical aspect of structural behavior. Moment redistribution in both normal-strength concrete (NSC) and continuous high-strength concrete (HSC) beams was the subject of investigation using nonlinear modeling. The findings of this study were subsequently validated through experiments conducted by the research by Lou et al.^21^. The research involved numerical modeling and experimentation on double-span continuous reinforced concrete beams with concrete strengths ranging from 30 to 90 MPa. The study focused on examining the impact of various ratios of tensile reinforcement across the middle support and upper spans on moment redistribution. It was observed that the degree of moment redistribution depended on the stiffness of critical sections within the beams. It is important to note that moment redistribution is closely associated with ductility.

In structures characterized by appropriate ductility, stress and moment redistribution within bending members occur at critical sections due to the development of plastic hinges, as highlighted by Mostofinejad and Farahbod^22^. Ehsani et al. investigated the estimation of the moment redistribution and plastic hinge characteristics in two-span beams cast with high-performance fiber-reinforced Cementitious composite. They concluded that increasing the transverse reinforcement had a positive effect on the moment redistribution compared with the reference beam, with significant differences in plastic hinge length^23^. The observed patterns of moment redistribution in experiments were juxtaposed with the criteria outlined in design codes, including the Structural Use of Concrete, Part 1: Code of Practice for Design and Construction from the British Standards Institution^24^. ACI 318 recommendations are excessively conservative^25^. In contrast, the values derived from the other code, namely Eurocode 2: Design of Concrete Structures Part 1 (2004), exhibit satisfactory conformity with experimental results ^26^. This comparative assessment was informed by both numerical and experimental investigations conducted on a total of 12 reinforced concrete (RC) beams, featuring two distinct depths of 400 mm and 720 mm, as documented in the research conducted by Yang et al.^27^. All of the beams in the study were characterized by similar configurations of top and bottom longitudinal reinforcement, and they were devoid of stirrups. The failure of these beams was primarily attributed to the development of diagonal cracks. Carmo and Lopez aimed to gain deeper insights into force redistribution capacity and other parameters of the beams^28^. This study explores the potential of using this material for conventional materials and evaluates parameters like load–displacement curves, energy absorption capacity, plasticity factors, ultimate strength, and moment redistribution.

Alva et al. tested concrete beam-column joints; the tested connections lacked slabs and had uniform reinforcement except within the joint area^29^. Nino Spinella introduced a model predicting shear strength^30^. In a separate study by Karimi and Hashemi, the feasibility of using steel fibers in concrete mixtures to strengthen opening sections in structures was explored as an alternative to intricate reinforcement detailing^31^. Moolaei et al. and Shao et al. experimentally demonstrated that HPFRCC enhances ductility, increases energy absorption, and modifies failure modes in beams^32,33^. Furthermore, Almeida et al. investigated plastic-hinge length and moment redistribution, highlighting how HPFRCC and stirrup spacing influence the development of the plastic hinge zone^34^.

## Research highlights

Experimental studies on beams with two fixed supports are limited in the literature; therefore, this paper investigates beams constructed with normal-strength concrete and High-Performance Fiber-Reinforced Cementitious Composite (HPFRCC). Owing to its high compressive and tensile strain capacity and strain-hardening behavior, HPFRCC is well-suited for earthquake-resistant flexural elements, such as fixed-end beams. The combined influence of steel fiber dosage and stirrup spacing enhanced moment redistribution, enabling similar ductility to normal concrete even with increased stirrup spacing. Overall, the use of HPFRCC significantly improved load capacity, energy absorption, ductility, and crack control, supporting the objectives of this study.

## Experimental plan

### Material properties

In this study, both normal concrete (NSC) and high-performance fiber-reinforced cementitious composite (HPFRCC) were developed through rigorous experimentation. The most effective mixing ratios, presented in Table 1, were determined based on weight. HPFRCC was designed with 1% and 2% steel fiber dosages, selected based on the authors’ previous research^23,32^. Portland cement (density: 3.05 g/cm^3^), sand, and gravel (particle diameters: 0.1 to 2.4 mm) were used in both HPFRCC and NSC. Silica fume (density: 2.2 g/cm^3^, grain size: 0.1 μm) was added, along with superplasticizer (density: 1.07 g/cm^3^), in concrete production. Table 2 outlines the properties of the reinforcing bars used in beam construction based on standard material tests. HPFRCC incorporated steel fibers (10 mm and 20 mm in length, 0.6 mm diameter) as reinforcing elements, meeting ASTM A 820 specifications (Table 3).

**Table 1:** Mixing scheme of normal and HPFRCC concrete.

Name of material	Cement (kg/m^3^)	Sand (kg/m^3^)	Gravel (kg/m^3^)	Water (kg/m^3^)	Superplasticizer (kg/m^3^)	Silica fume (kg/m^3^)	Steel fiber content by volume (%)
Normal	450	597	1083	210	-	-	-
HPFRCC	850	1062	-	257	13.77	85	1 &2

**Table 2:** Steel bars specifications.

Diameter $\Phi$ (mm)	Type of bar	Yielding strength (ƒ_y_) (MPa)	Ultimate strength (ƒ_u_) (MPa)	Yielding strain (ɛ_sy_)	Modulus of elasticity (E_s_) (GPa)	Ultimate strain (ɛ_su_)
16	Longitudinal bar	546	672	0.0025	210	0.16
12	Longitudinal bar	556	696	0.0024	210	0.16
8	Transverse bar	535	800	0.0020	210	0.14

**Table 3:** Steel fibers characterization.

Type of fiber	Length, l (mm)	Diameter, d (mm)	Aspect ratio $\left( \frac{l}{d} \right)$	Tensile strength (MPa)	Modulus of elasticity (GPa)	Density $\left( {\frac{{{\text{kN}}}}{{{\text{m}}^{3} }}} \right)$
Hooked-end	10	0.6	16.67	1100	200	78

**Fig. 1:**
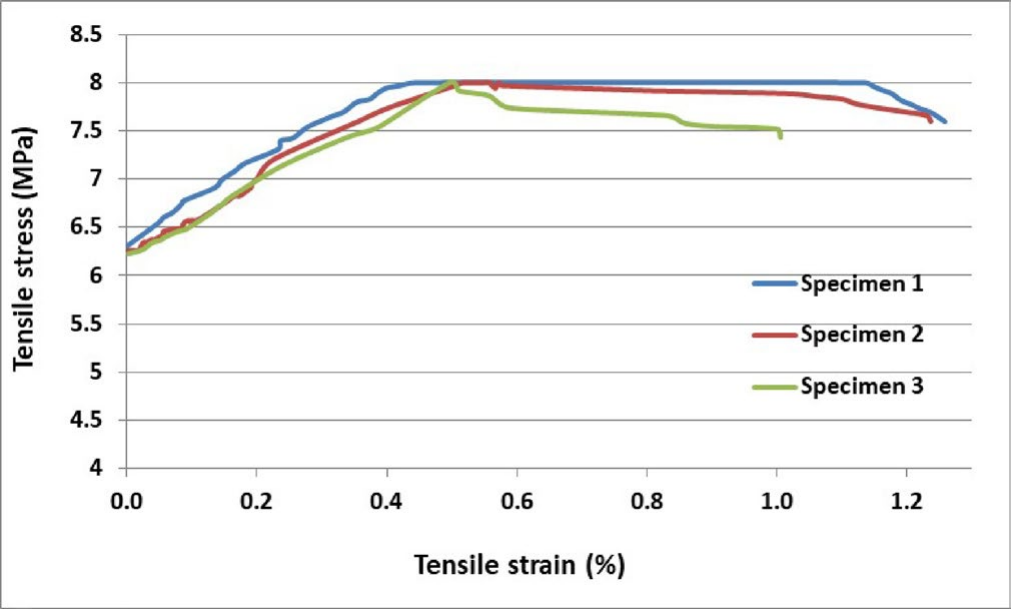
Stress–Strain relationship in 2 volume percentage of fibers.

Experimental beams were made from both normal and HPFRCC concretes, with 150 mm compressive cubic samples. The average compressive strength for normal and HPFRCC concrete cylinder specimens was considered to be 55.7 and 60.4 MPa, respectively. The average density of both concretes was 22.23 kN/m^3^. To analyze HPFRCC’s mechanical performance based on fiber volume, a dog bone tensile test was conducted using the apparatus. Notably, tested specimens exhibited a maximum ultimate tensile stress of up to 6.2 MPa and a strain of 1.3% from four specimens, as indicated in Table 4 and Fig. 1. The ultimate strain was measured after a 10% degradation of ultimate stress.

**Table 4:** Uniaxial tension test results for HPFRCC specimens.

Sample	First crack stress (MPa)	First crack strain (%)	Ultimate stress at the peak (MPa)	Ultimate strain after 10% degradation of ultimate stress (%)
1	4.07	0.02	6.2	1.3
2	3.93	0.02	5.9	1.27
3	3.77	0.02	5.6	1.20
4	3.77	0.01	5.7	1.10

### Experiment’s process

**Fig. 2:**
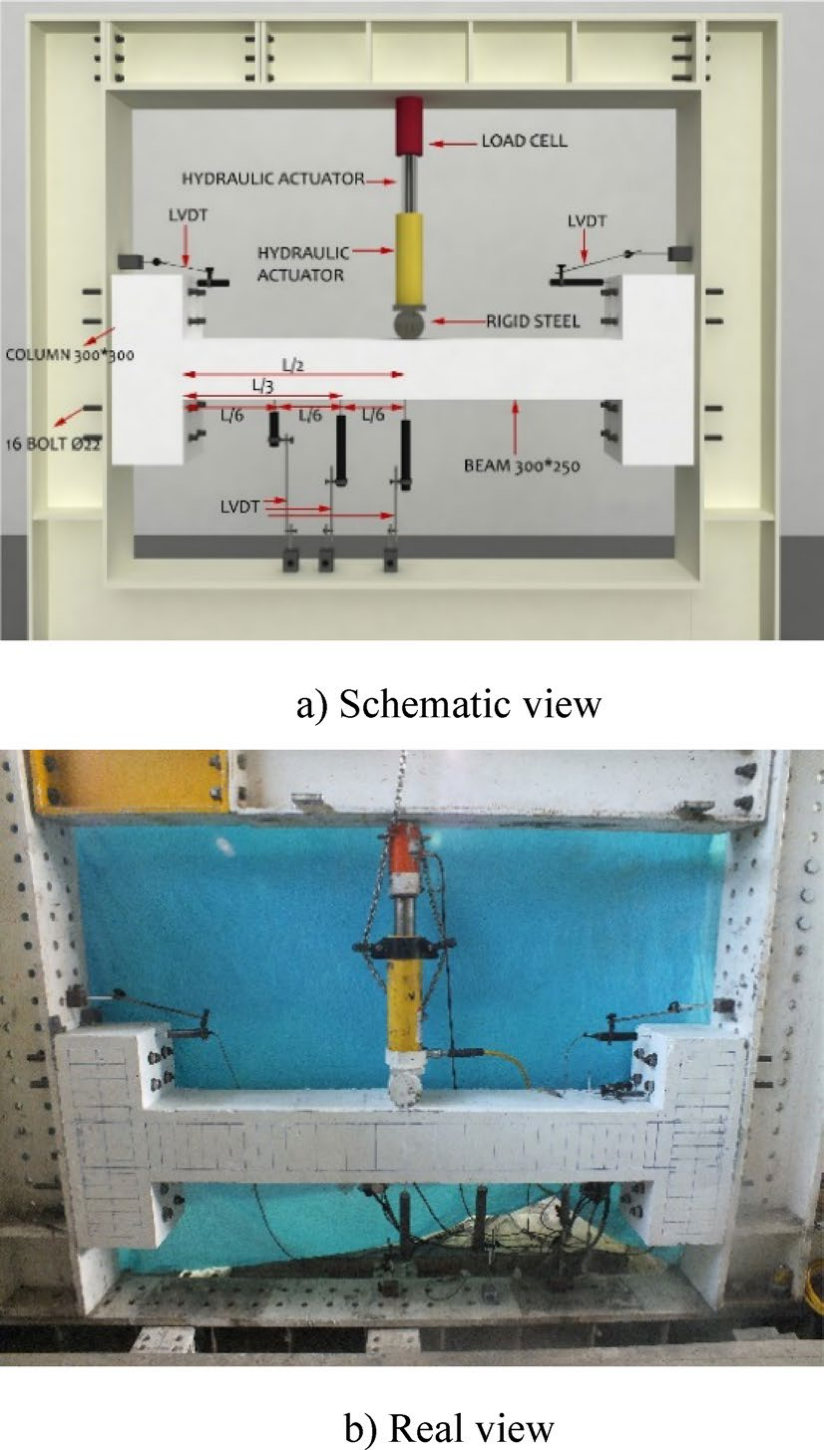
Experiment’s setup for one-point Load testing specimens.

**Fig. 3:**
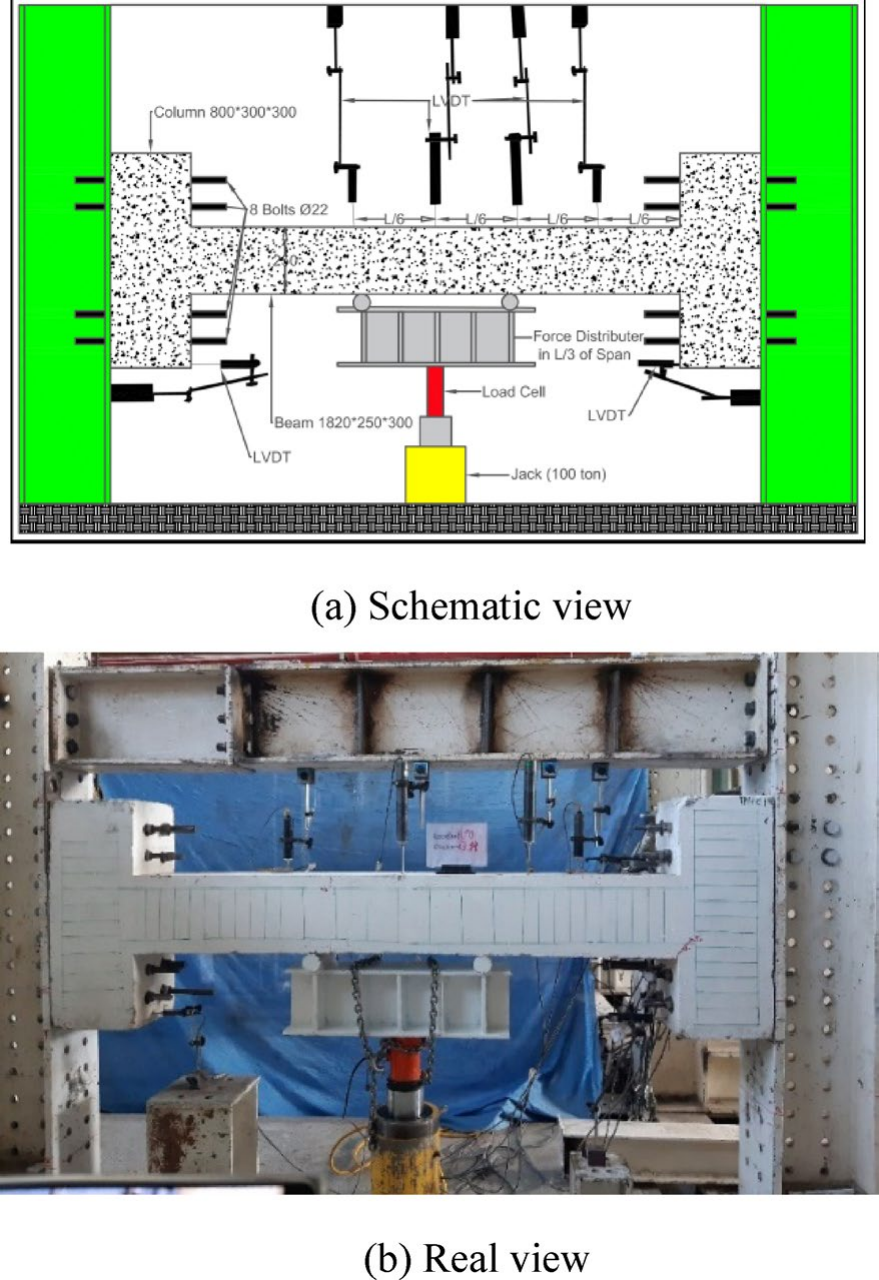
Experiment’s setup for two-point Load testing specimens.

To explore the characteristics of beams with fixed supports, a total of 8 beams in two groups were taken into consideration, as outlined in Table 5. Among the eight beams examined, two served as reference beams made of normal concrete (N). One of these reference beams featured closely spaced, NC (one-fourth of effective height, d/4, as recommended by design codes, 8-mm bar at spacing 50 mm) stirrups within the plastic zone, while the other had wide (Not closely spaced) stirrups, NN (half of effective height, d/2, 8-mm bar at spacing 100 mm). Additionally, there were two beams reinforced with 1% steel fibers, each having one with closely spaced stirrups, HC1, and the other with non-closed stirrups, HN1. Furthermore, two concrete beams were fabricated with 2% steel fiber content, with one having closely spaced stirrups, HC2, and the other featuring non-closed stirrups, HN2. All six of these beams, as Group 1, were subjected to a static, one-point load applied at the midpoint of the beam, as shown in Fig. 2. The remaining two concrete beams, as Group 2, containing 1% steel fibers, 2PHN1, and 2PHC1, were subjected to a two-point load positioned at one-third of the span, as shown in Fig. 3.

**Table 5:** The characteristics of concrete specimens.

Group	Specimen notation	Longitudinal bars		Stirrups of the beam		Applied load location
		Top bars (Negative moment side)	Bot bars (Positive moment side)	At critical zone	At the middle	
1	NN^1^ HN1^2^ HN2^3^	2 $\emptyset$ 12 2 $\emptyset$ 16	2 $\emptyset$ 12 3 $\emptyset$ 16	$\emptyset 8@100{\text{ mm}}$ (d/2)	$\emptyset 8@100$ mm	Middle
	NC^4^ HC1^5^ HC2^6^	2 $\emptyset$ 12 2 $\emptyset$ 16	2 $\emptyset$ 12 3 $\emptyset$ 16	$\emptyset 8@50{\text{ mm}}$ (d/4)	$\emptyset 8@100$ mm	
		Top bars (Positive moment side)	Bot bars (Negative moment side)			
2	2PHN1^7^	2 $\emptyset$ 12 2 $\emptyset$ 16	2 $\emptyset$ 12 3 $\emptyset$ 16	$\emptyset 8@100{\text{ mm}}$ (d/2)	$\emptyset 8@100$	One-third
	2PHC1^8^			$\emptyset 8@50{\text{ mm}}$ (d/4)	$\emptyset 8@100$	One-third

^1^NN Specimen with Normal concrete and not close stirrup (wide-spacing).

^2^HN1 Specimen with 1% fiber-HPFRCC concrete and not close stirrup (wide-spacing).

^3^HN2 Specimen with 2% fiber-HPFRCC concrete and not close stirrup (wide-spacing).

^4^NC Specimen with Normal concrete and Close stirrup (close-spacing).

^5^HC1 Specimen with 1% fiber-HPFRCC concrete and Close stirrup (close-spacing).

^6^HC2 Specimen with 2% fiber-HPFRCC concrete and Close stirrup (close-spacing).

^7^2PHN1 Specimen under 2-Point loading with 1% fiber-HPFRCC concrete and not close stirrup (wide-spacing).

^8^2PHC1 Specimen under 2-Point loading with 1% fiber-HPFRCC concrete and Close stirrup (wide-spacing).

Therefore, the Load is applied incrementally at midspan (NN, NC, HC1, HN1, HN2, HC2) and one-third span (2PHN1, 2PHC1) to the beams. The length of the beams was 1820 mm, and the total length of the elements was 2420 mm. A 1000 kN hydraulic jack applied concentrated loads as shown in Figs. 2 and 3, with a corresponding load cell measuring the force. Beam deflection was tracked using LVDTs at three positions: midpoint, 0.333 span length, and 0.666 span length.

**Fig. 4:**
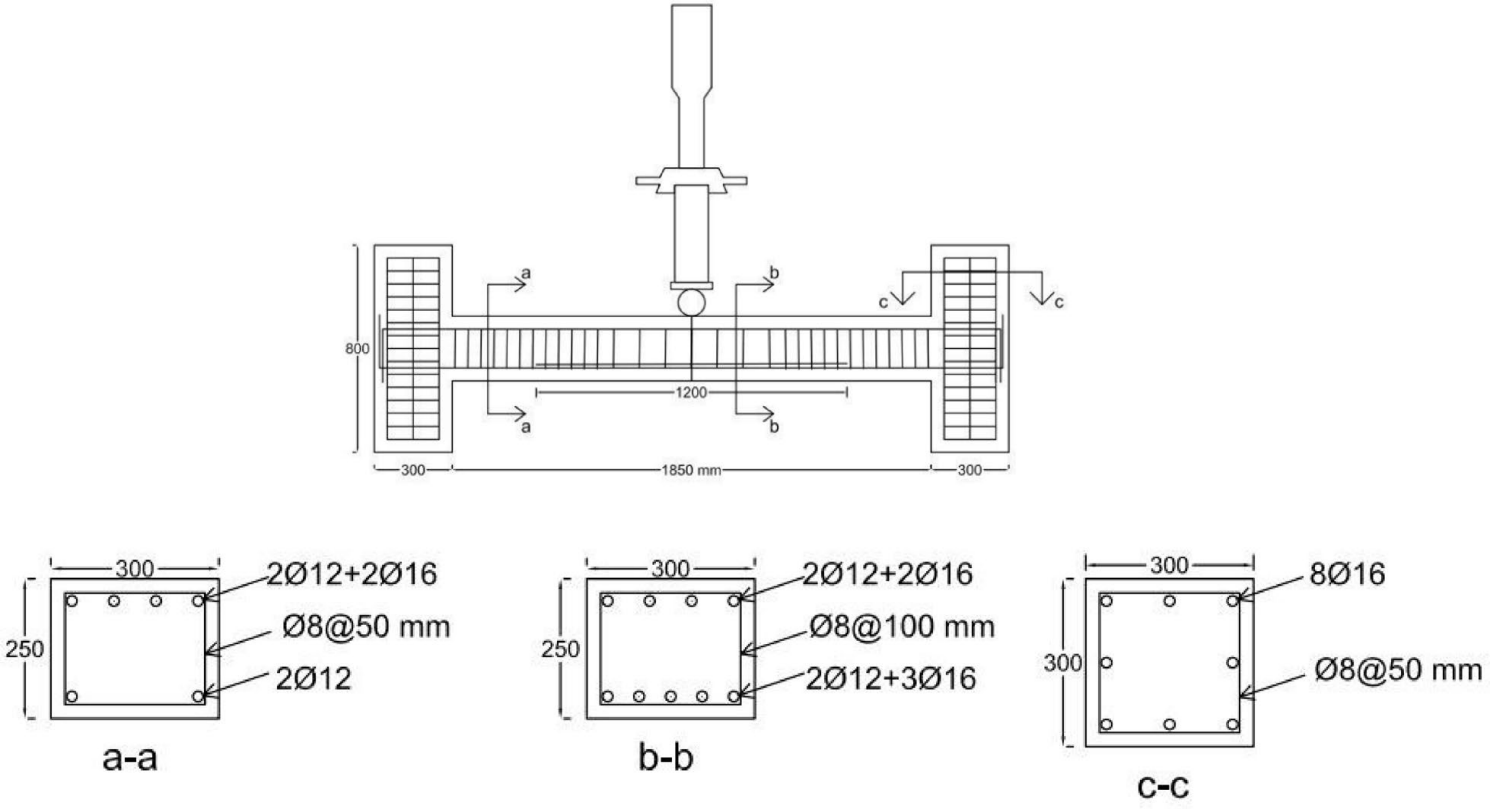
Specimens’ properties.

After conducting the experiments with point loading according to Fig. 3, the actual capacities of the beams were found to be approximately double the estimated capacities based on the initial relationships from ACI 318, considering the effects of bar stiffening and redistribution of anchor forces. Very small displacements were observed in the rigid beam, causing minor damage to the rigid beam and the testing system. Therefore, the experimental setup was necessarily rearranged to ensure consistent conditions, following the initial configuration as shown in Fig. 3. The concentrated loading was applied from the bottom (rigid base) to the top by rotating the specimens 180 degrees. Due to the consistent conditions, the results are examined and compared with minimal errors. Beam dimensions were 300 × 250 mm (width, b = 300, and total height, h = 250. and effective height, d = 200 mm), connected to the frame via 300 × 300 mm columns with a total of 16 grade-20 bolts (8 bolts on each side) (Fig. 4). Beams subjected to a concentrated load at the middle have the same moment at the support (negative moment) and at the middle of the beam (positive moment). To prevent hinges from forming simultaneously at the midspan and supports, the positive moment capacity must exceed the negative moment capacity. For this reason, the beams were detailed with continuous reinforcement consisting of two 12 mm bars and two 16 mm bars at the top, two 12 mm bars at the bottom, and three additional 16 mm bars at the midspan bottom.

**Fig. 5:**
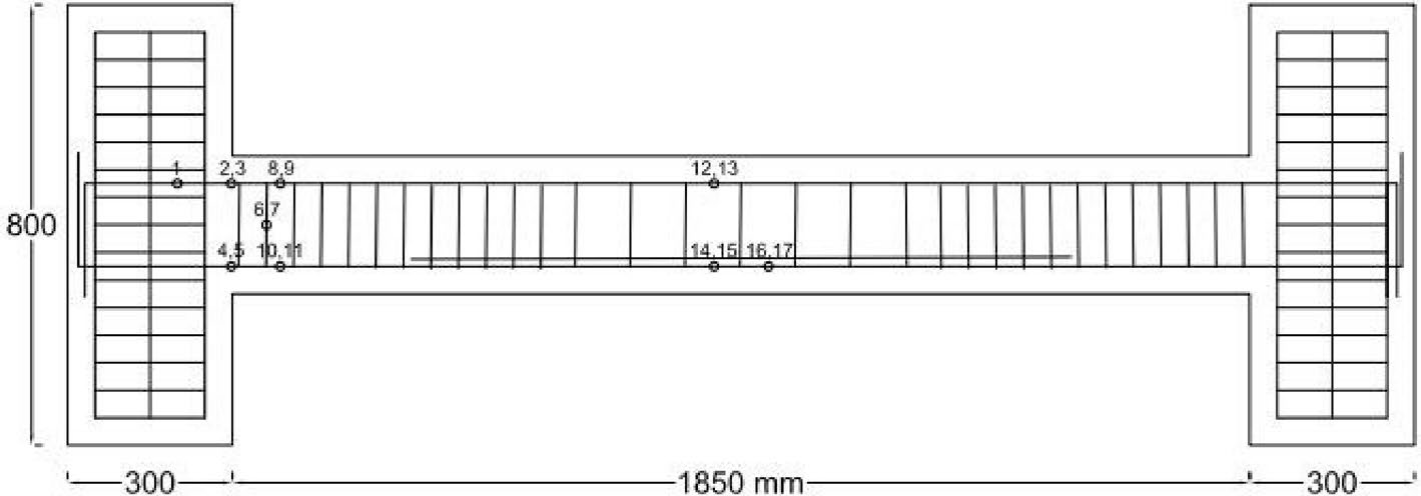
Strain gauge’s location on steel bars.

Electrical strain gauges (ESG) of 10 mm dimensions and 120 Ohms resistance were attached at 17 points on longitudinal bars and stirrups for strain measurement (Fig. 5). The beams, supported at both ends, had a central span of 1.85 m, short columns on each side, and met fixed-end conditions ensured by 16 bolts and constant monitoring.

## Analyzing the experimental results

During experimental testing of beams under concentrated (one-point) or two-point loads, various aspects such as failure patterns, toughness, and energy absorption are studied. The results highlight that crack initiation and propagation depended on material properties, concrete tensile strength, and reinforcement type. Typically, initial cracks appeared at the midpoint in all beams, showing a flexural nature. Normal concrete beams exhibit consistent flexural and flexural-shear cracks, extending towards supports and short columns. In high-performance beams, small flexural cracks develop near the supports and midpoints, eventually leading to structural failure as they widen.

### Load–displacement curve

**Fig. 6:**
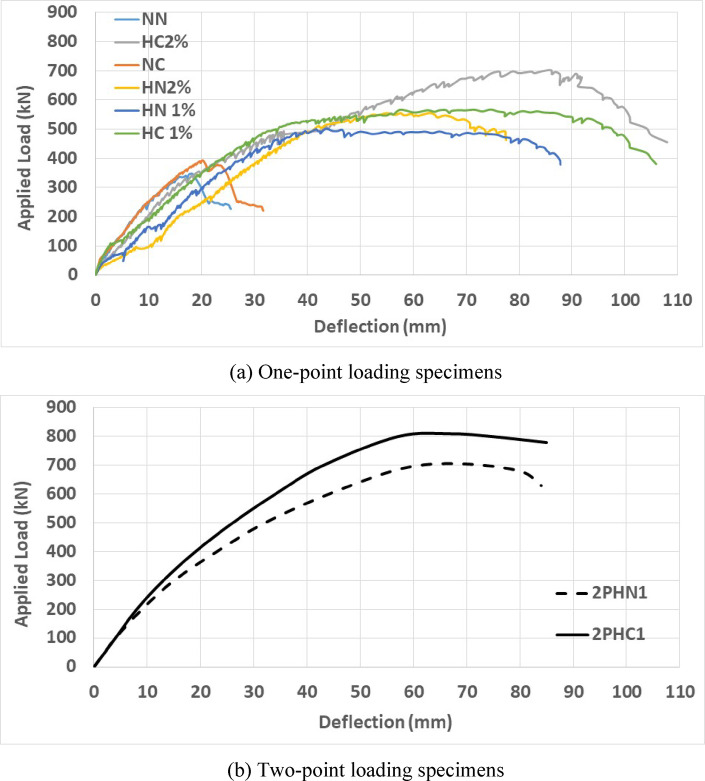
Load–Displacement curves of specimens.

Maximum loads (${P}_{max}$, peak loads), recorded using load cells for each beam (as detailed in Tables 6 and 7 and Fig. 6, represent the highest quantified loads. The equivalent load at the first observed crack is ${P}_{cr}$ while ${P}_{y}$ as the yielding load obtained from a strain gauge reading instrumented on steel bars at the yielding point. Ultimate load, ${P}_{u}$, is approximately at 0.85 times of maximum load with corresponding displacement ${\Delta }_{u}$. By observing and analyzing the initial results, it is noted that the initial part of the load-deformation curve of the specimens is linear. After cracking, the stiffness decreases during the load increment. Different gained loads and corresponding displacements can be observed in Tables 6 and 7. It is noticed that the use of more stirrups in the plastic zone has a limited effect on the cracking load, but somewhat reduces the displacement changes associated with cracking. This phenomenon is observed in both normal concrete and 1% and 2% fiber-reinforced concrete.

**Table 6:** Loads details of the tested beams.

Group	Specimen notation	Cracking load	Yielding load (Strain-Guage)	Increasing ${P}_{y}$ compare to ${P}_{y\left(N.N\right)}$ (%)	Maximum load	Increasing ${P}_{max}$ compare to ${P}_{max\left(N.N\right)}$ (%)	Failure mode
		${P}_{cr}$ (kN)	${P}_{y}$ (kN)		${P}_{max}$ (kN)		
Group 1	NN	20	257	-	345	-	Flexural-Shear
	NC	30	303	17	393	14	Flexural-Shear
	HN1	45	347	35	498	44	Flexural
	HC1	45	400	55	566	64	Flexural
	HN2	50	370	44	552	61	Flexural
	HC2	50	450	75	702	104	Flexural
Group 2				${P}_{y\left(2PHN1\right)}$		${P}_{max\left(2PHN1\right)}$	
	2PHN1	50	480	-	705	-	Flexural
	2PHC1	70	500	4	810	14	Flexural

**Table 7:** Displacements detail of the tested beams.

Group	Specimen notation	Cracking load	Increasing ${\Delta }_{cr}$ compare to ${\Delta }_{cr\left(N.N\right)}$ (%)	Yielding load	Increasing ${\Delta }_{y}$ Compare to ${\Delta }_{y\left(N.N\right)}$ (%)	Ultimate load	Increasing ${\Delta }_{u}$ compare to ${\Delta }_{u\left(N.N\right)}$ (%)
		${\Delta }_{cr}$ (mm)		${\Delta }_{y}$ (mm) (Strain-Guage)		${\Delta }_{u}$ (mm)	
Group 1	NN	0.42	-	10.5	-	21	-
	NC	0.74	76	13.17	25	28	33
	HN1	1.54	266	23	119	85	305
	HC1	1.85	340	22.6	115	98	366
	HN2	3.57	750	24	128	77	266
	HC2	2.56	510	26	150	95	352
Group 2			${\Delta }_{cr\left(2PHN1\right)}$		${\Delta }_{y\left(2PHN1\right)}$		${\Delta }_{u\left(2PHN1\right)}$
	2PHN1	1.45	1	23.5	1	85	1
	2PHC1	3.14	116	18.5	1	84	1

- **Stirrup spacing effect**

The maximum applied load of NN was 345 kN, with a corresponding 18.34 mm displacement. Conversely, NC tolerated more load and deflection. Reducing stirrup spacing from d/2 of the NN specimen to d/4 of the NC specimen led to an approximately 14% increase in P_max_. In closed spacing beams, the ultimate displacement increased by 33%, emphasizing stirrup spacing’s greater influence on deformation compared to load enhancement. The HN1 specimen had a maximum applied load of 498 kN and a 44.1 mm displacement, but the P_max_ of HC1 was 566 kN, with a 75 mm displacement. Reducing stirrup spacing at 1% fiber volume leads to a 20% increase in P_max_ and a significant 61% increase in ultimate displacement. HC2 specimen showed a substantial 43% increase in maximum load and a significant 86% increase in corresponding ultimate displacement compared to wide-spacing stirrup beams (HN2). The 2PHC1 specimen subjected to two-point loading shows a substantial 14% increase in maximum load without a significant change in corresponding ultimate displacement compared to wide spacing stirrup beams 2PHN1. The deformation-load behavior exhibits linearity before cracking, called ${P}_{cr}$, followed by a decreasing stiffness trend as the load increases up to the yielding point, called ${P}_{y}$. Reducing stirrup space is more effective on cracking load and displacement of beams without fiber. Yielding loads and displacements of beams are average, and increase by 25% by reducing the stirrup spacing.

- **Steel fiber effects**

Introducing 1% fibers to normal concrete with wide-spaced stirrups (HN1) leads to a remarkable 44% increase in P_max_ and a significant 305% increase in corresponding ultimate displacement when compared to regular concrete (NN). Similarly, in high-performance concrete with 2% fiber (HN2), this change results in a 61% increase in P_max_ and an impressive 266% increase in ultimate displacement. Increasing the fiber percentage from 1 to 2% in HPFRCC concrete leads to improved capacity and displacements.

Increasing 2% fiber to wide-spaced stirrups (HN2) leads to a 17% increase in P_max_ and a 40% decrease in corresponding ultimate displacement due to more stiffness when compared to the specimen with 1% fiber (HN1). The maximum load of closed-spaced stirrups (HC2) is increased by 40% by increasing fiber percentage from 1 to 2% fiber when compared to the specimen with 1% fiber (HC1), with a negligible change in the corresponding ultimate displacement. Therefore, these results highlight significant improvements in load capacity and ductility by adding fiber to the mix design. Increasing fiber is more effective in cracking and yielding loads and displacements of the tested beams. Cracking loads and displacement are increased by 1 and 2% fibers up to 2 and 8 times compared to specimens without fibers, respectively. Moreover, yielding loads and displacement are increased by adding fibers up to 75 and 150% compared to specimens without fibers, respectively.

### Crack patterns of specimens

**Fig. 7:**
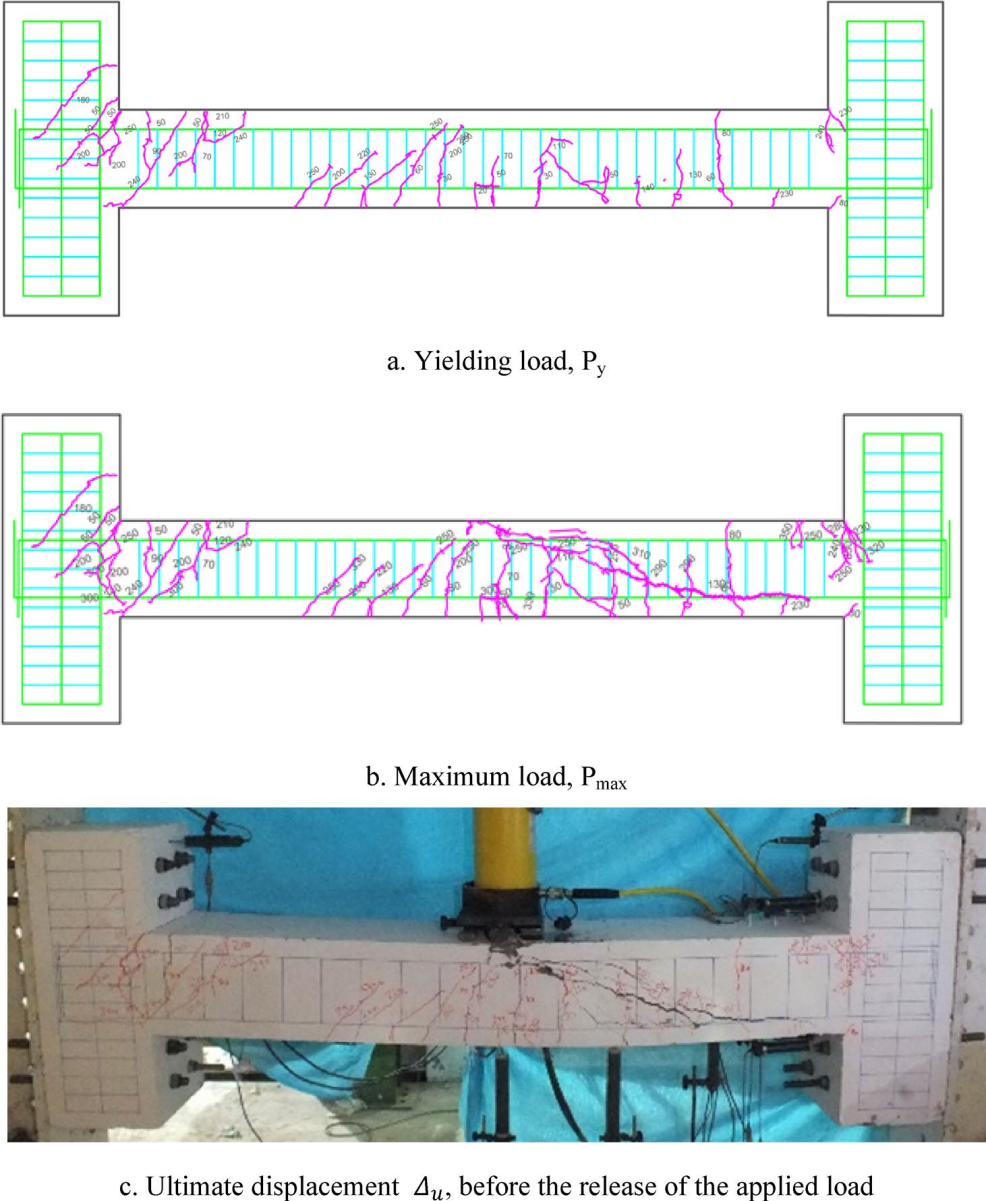
Failure shape and cracking pattern of the NN beam subjected to the middle point load.

**Fig. 8:**
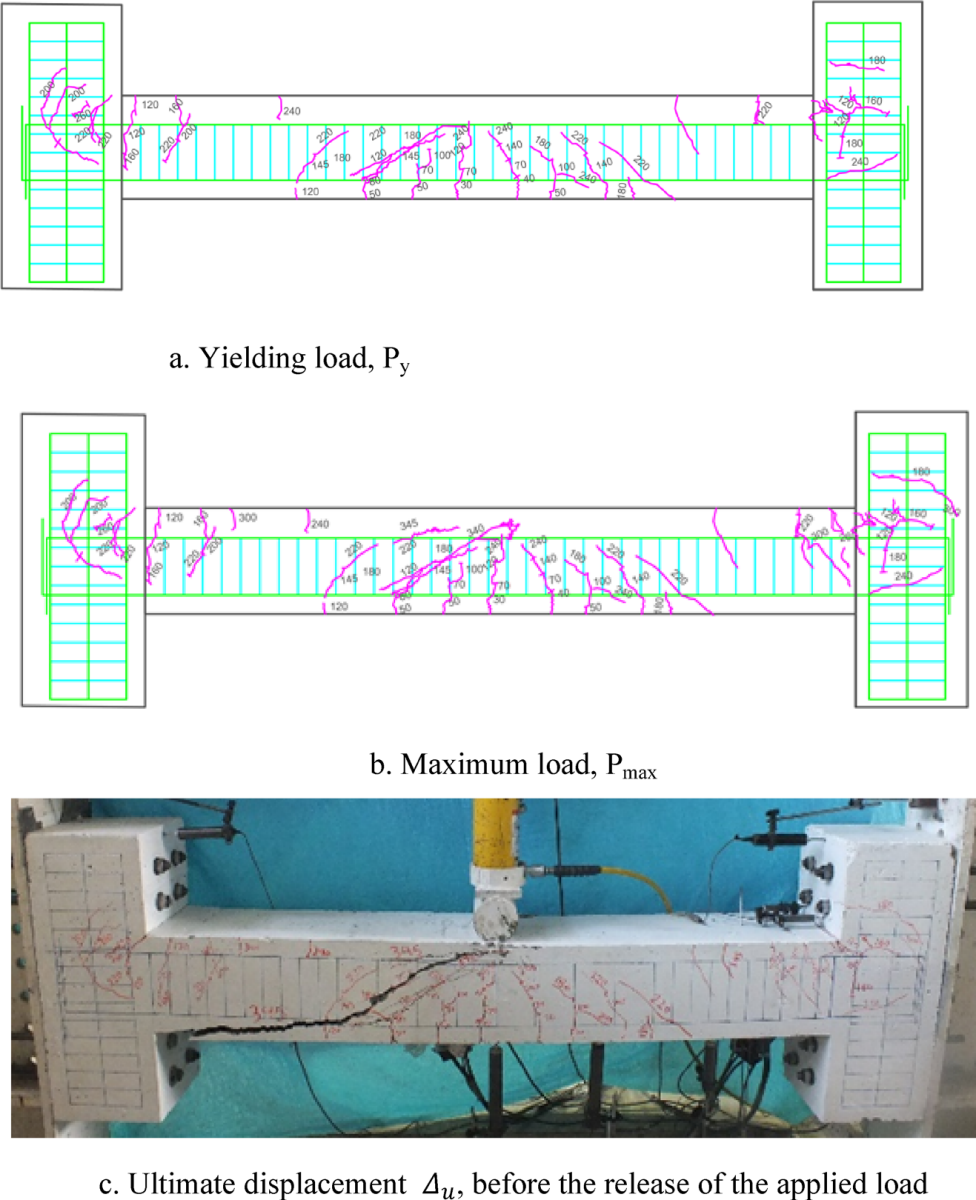
Failure shape and cracking pattern of the NC beam subjected to the middle point load.

**Fig. 9:**
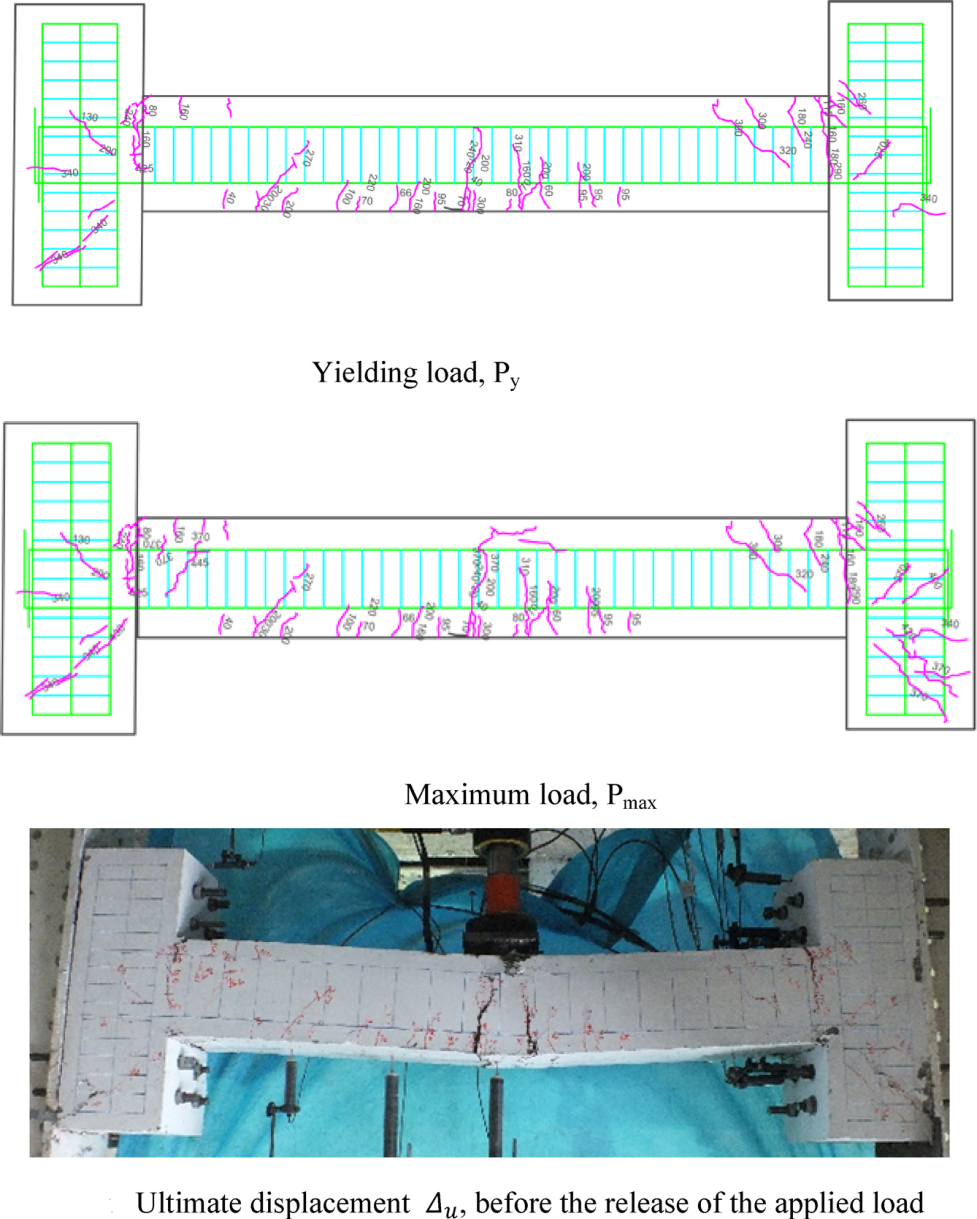
Crack pattern of the HN1 beam subjected to the middle point load.

**Fig. 10:**
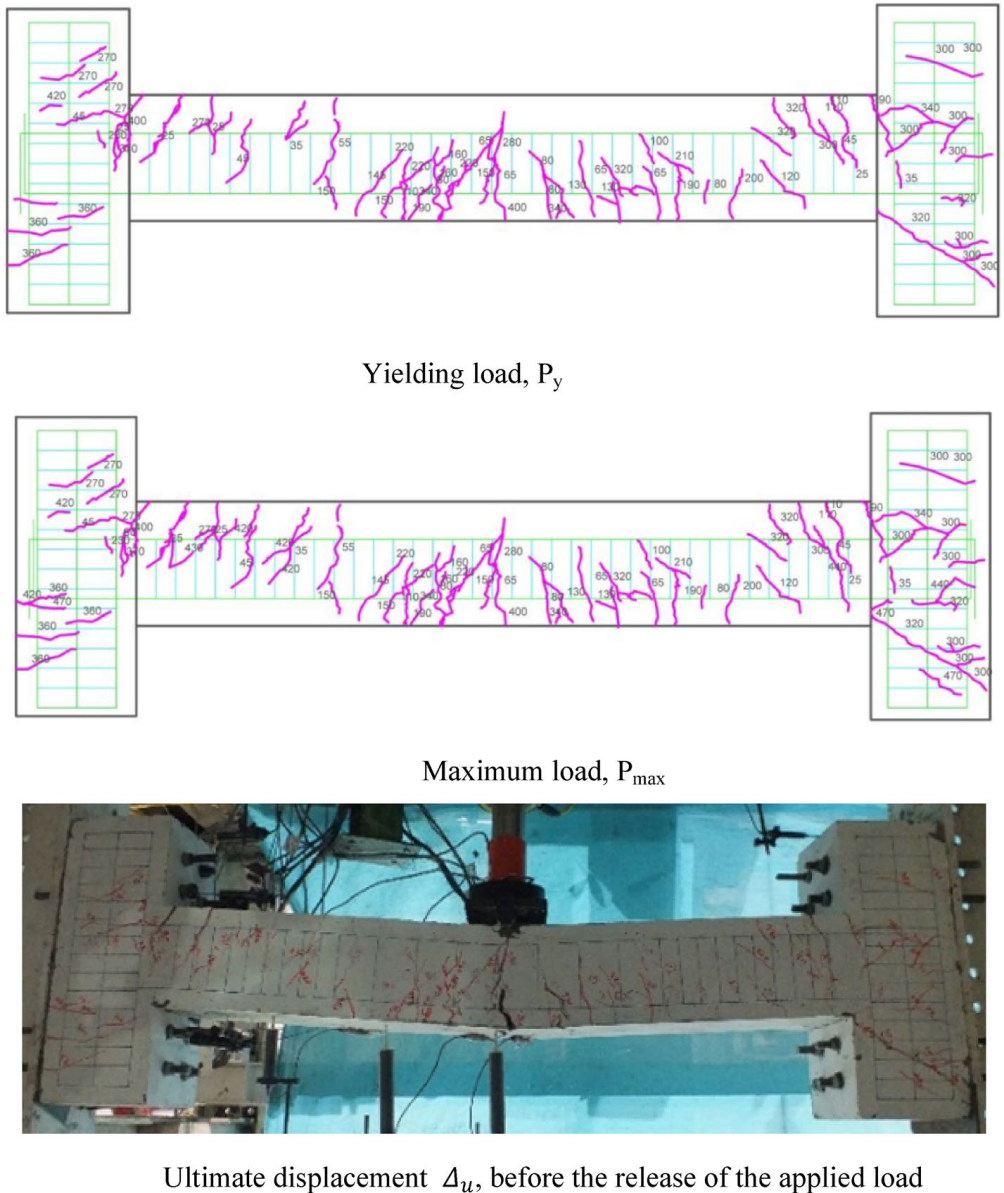
Failure shape and cracking pattern of the HC1 beam subjected to the middle point load.

**Fig. 11:**
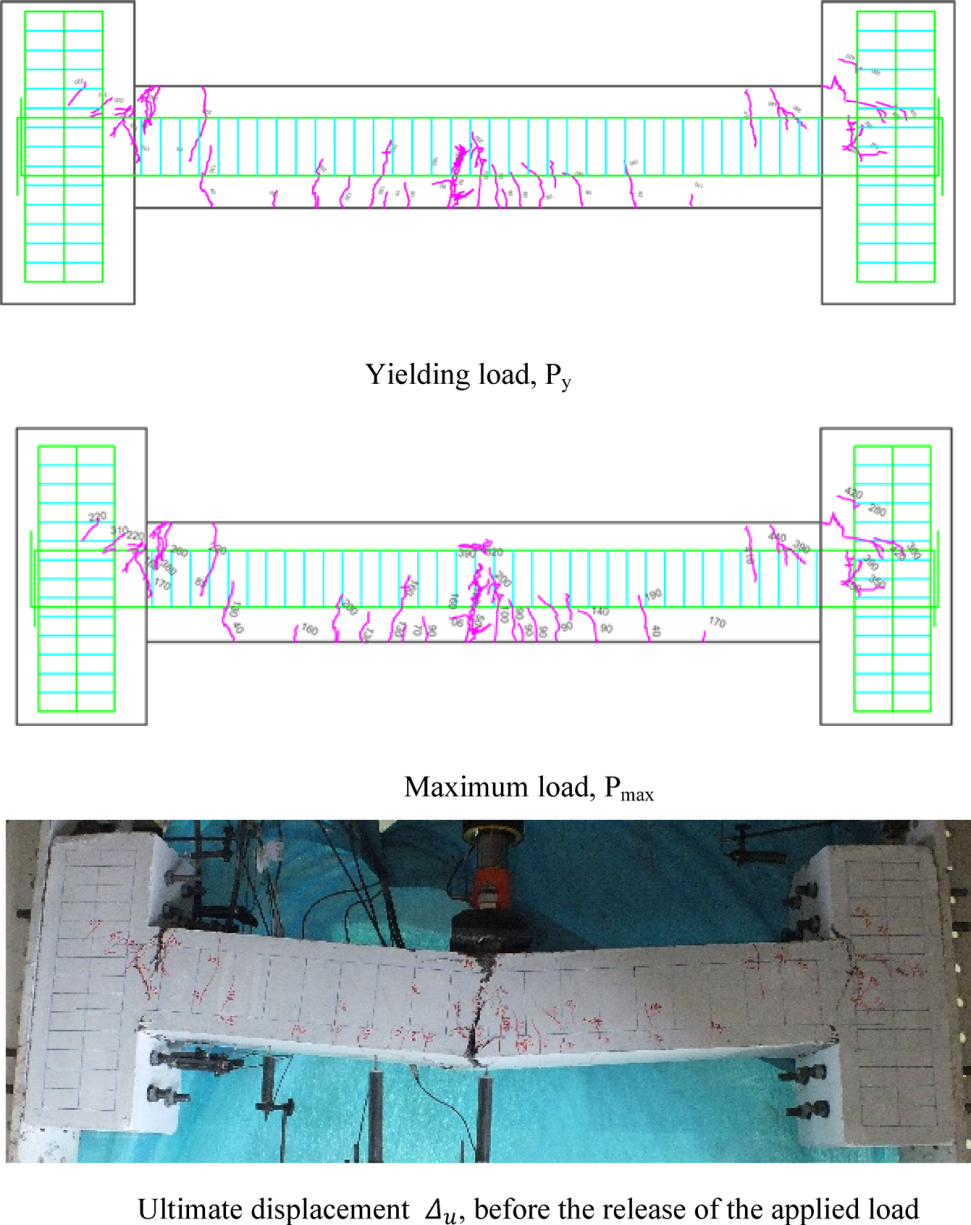
Failure shape and cracking pattern of the HN2 beam subjected to the middle point load.

**Fig. 12:**
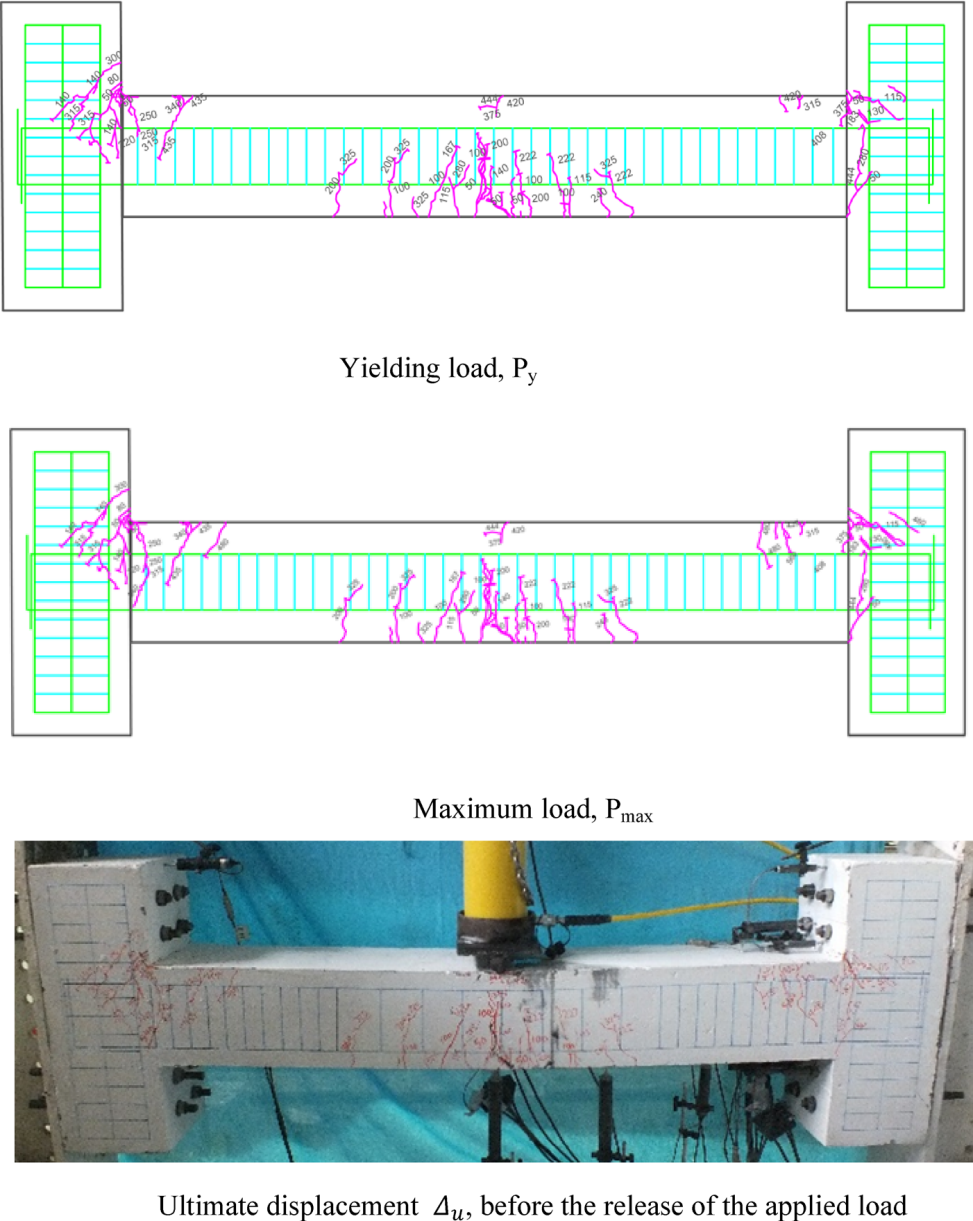
Failure shape and cracking pattern of the HC2 beam subjected to the middle point load.

**Fig. 13:**
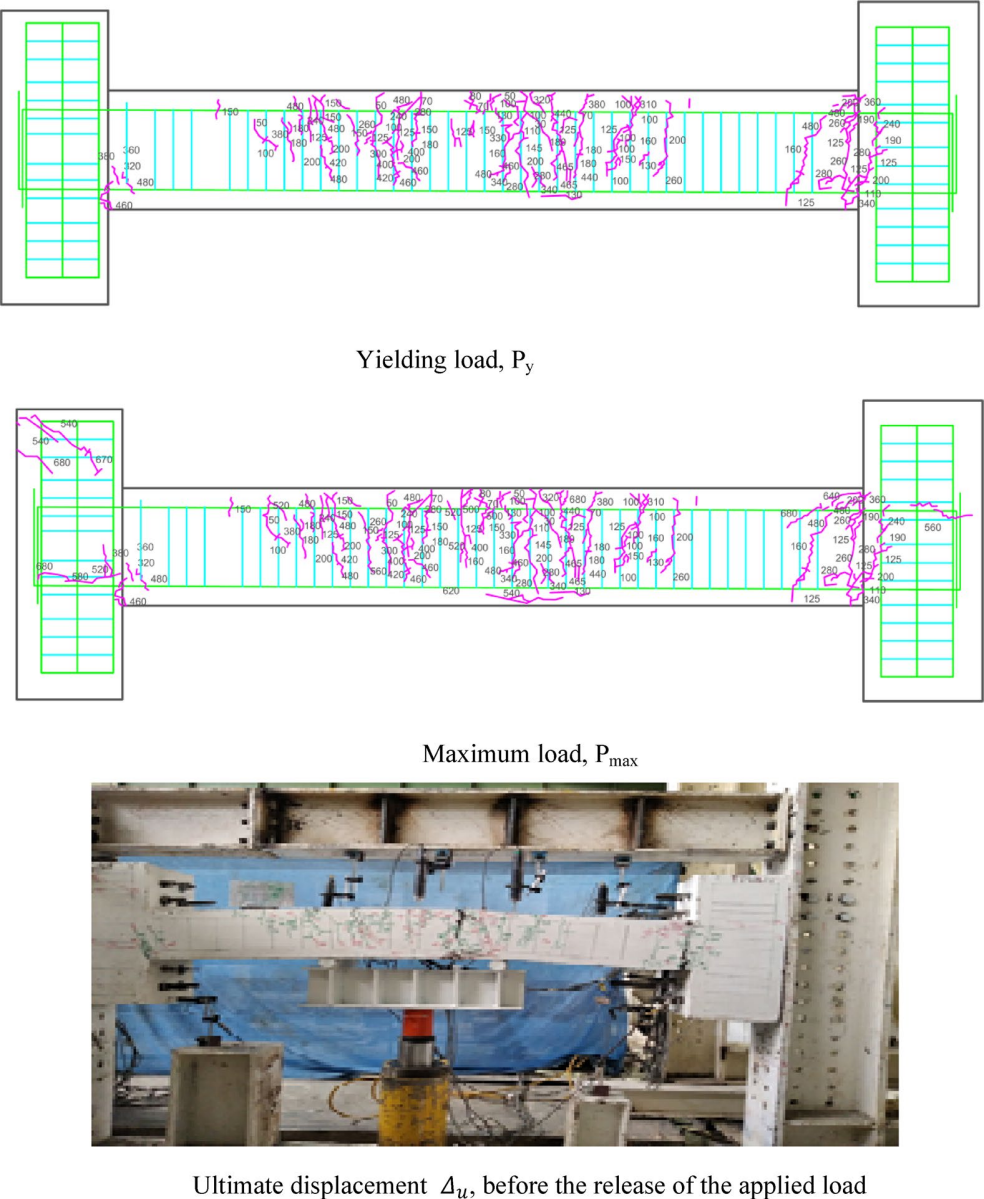
Failure shape and cracking pattern of the 2PHN1 beam subjected to two-point load.

**Fig. 14:**
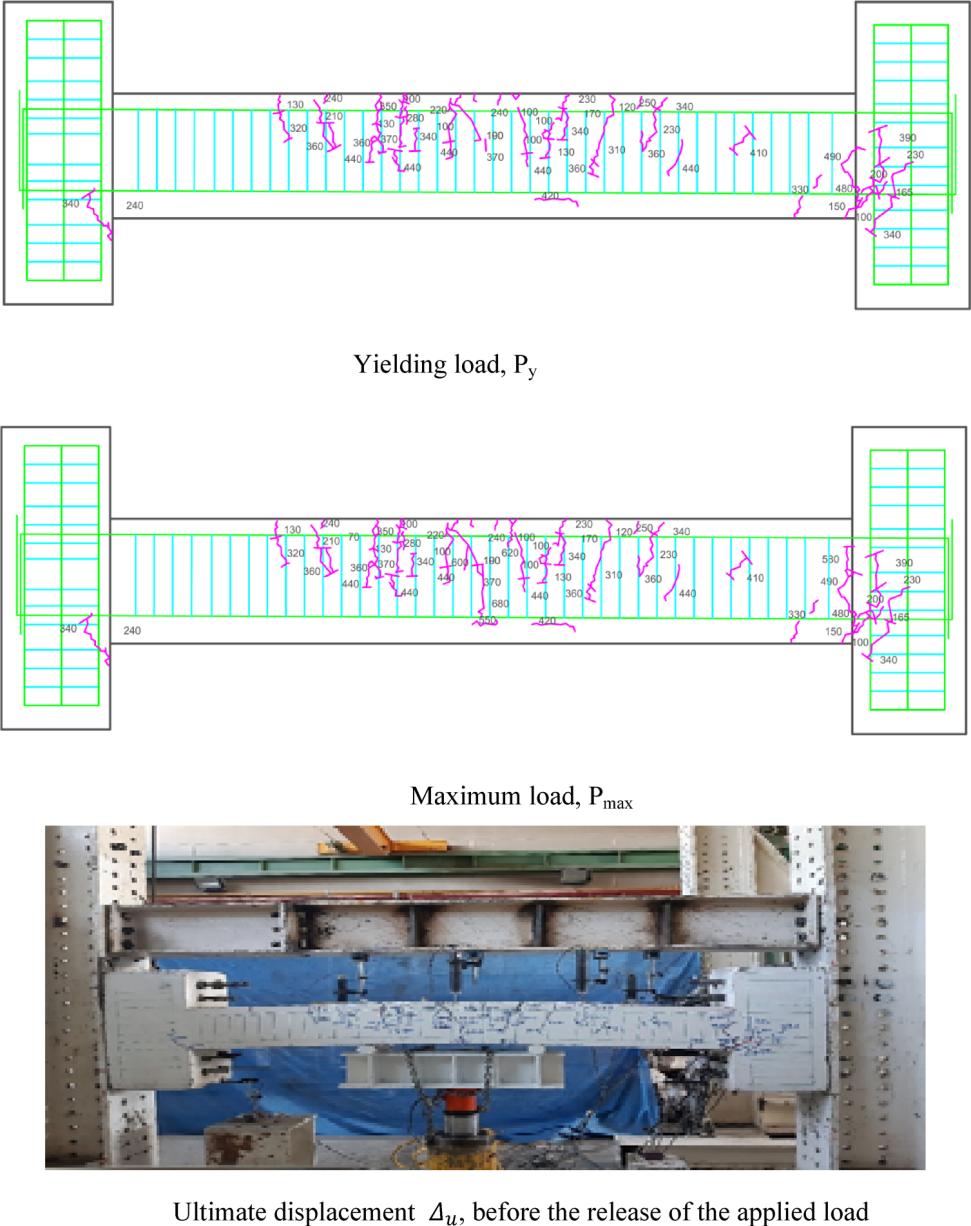
Crack pattern of 2PHC1 beam subjected to two-point load.

Figures 7, 8, 9, 10, 11, 12, 13 and 14 present the schematic and actual crack patterns observed in specimens. These cracks are documented at different stages of loading, including the yielding load (P_y_), maximum load (P_max_), and ultimate displacement leading to collapse (Δ_u_). In the initial phase, flexural cracks emerge at the midpoint of the span in all tested beams. In regular concrete beams, these cracks propagate toward the load point and extended into the compressive region of the support.

Ultimately, the widening of these cracks led to beam failure. In contrast, high-performance beams exhibit a higher number of flexural cracks that extend toward the load point as the load increased. Subsequently, cracks in the middle of the beam widened, ultimately failing. Figures 7, 8, 9, 10, 11, 12, 13 and 14 demonstrate that high-performance concrete beams, especially those reinforced with spaced stirrups, displayed exceptional ductility and significantly reduced crack width. Additionally, an increase in fiber volume fraction leads to more cracking, with most cracks initiating at higher loads. This phenomenon occurred because the fibers delayed crack development and widening. In the control beams without fibers (depicted in Figs. 9 and 10), steel bars yield at 257 and 303 kN before concrete was crushed on both sides of the support. Major shear cracks extend until reaching P_max_, after which the initial cracks widened, and new minor cracks formed up to P_u_ equal to 15% degradation after maximum load. This pattern indicated the initiation of beam failure with the main diagonal shear crack originating from the middle of the beam towards the support. Notably, the NC specimen displayed more cracks with less angled main diagonal cracks due to its flexible and ductile behavior, attributed to the closely spaced stirrups in the critical zone at the ends of the beam.

In the HN1 and HC1 beams, both containing 1% fiber (as depicted in Figs. 9 and 10), steel bars yielded at 347 and 400 kN, respectively, before concrete crush occurred on both sides of the support. At the midpoint, flexural cracks widen, leading to the eventual failure of the beams. While the HN1 specimen displayed limited minor cracks, the HC1 specimen exhibited more cracks extending up to the ultimate displacement, highlighting the greater influence of higher fiber content. Notably, no diagonal cracks were observed in these fiber-reinforced specimens, even up to the failure modes, indicating a failure pattern dominated by flexure. Similarly, the HN2 and HC2 specimens, both containing 2% fiber, exhibited the same flexure-dominated failure behavior and crack patterns. However, the HC2 specimen, reinforced with closely spaced stirrups, shows higher yielding and maximum capacity. Diagonal shear-dominated cracks at the middle or corners were absent in both specimens. When comparing the two specimens subjected to two-point loading, they exhibit the same yielding capacity but with different crack patterns. Specimen 2PHN1, with wide spacing stirrups, displays more initial cracks, indicating greater initial damage. Additionally, the maximum capacity of 2PHC1, with closed spacing stirrups, surpass that of the wide spacing stirrup counterpart and exhibited fewer final cracks, underscoring the enhanced effectiveness of 2% fiber compared to 1% fiber in achieving maximum capacity and minor cracks, crucial for potential specimen repairs. Both specimens exhibit a flexure-dominated behavior without significant shear diagonal cracks at the supports.

### Ductility

**Fig. 15:**
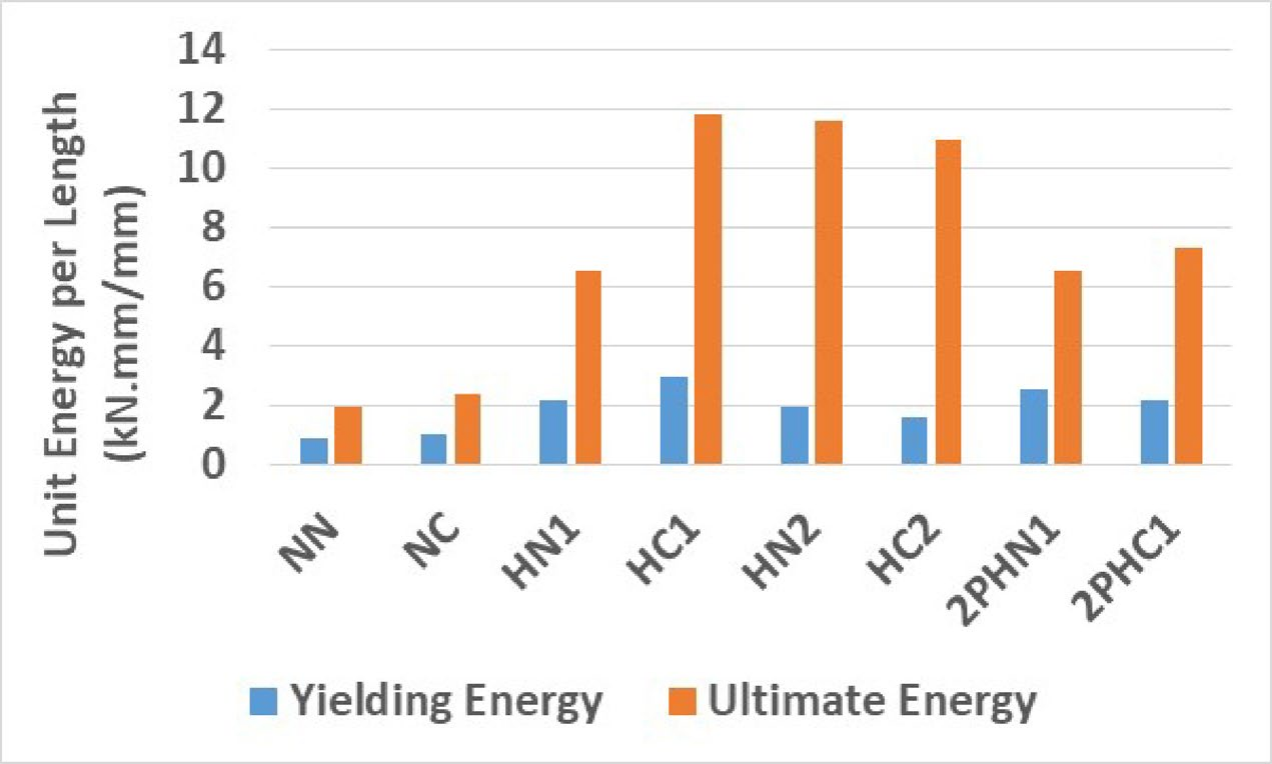
The normalized values of unit energy per length of all specimens.

Beam ductility, crucial in indeterminate structures like continuous and fixed beams, refers to the ability to deform inelastically without losing its load-carrying capacity until failure. Ductility is often measured using the ductility factor (μ), comparing rotations (θ), curvatures (φ), displacements (Δ), and absorbed energy (E) at failure to their values at yielding. The displacement ductility factor (μ_∆_), represented as μ_∆_ = ∆_u_/∆_y_ (where ∆u is the final deformation and ∆_y_ is the yield deformation at the center of the beam for the tensile bar), quantifies ductility. Table 8 shows increased ductility with reduced stirrup spacing and concrete fibers, as observed. Another approach uses the energy index (μ_E_ = E_u_/E_y_), comparing energies absorbed at ultimate load (E_u_) and yield load (E_y_). Both methods indicate enhanced ductility with fiber inclusion. The normalized values of yielding and ultimate energy per length in term of kN.mm/mm of all specimens are drawn and shown in Fig. 15. The results indicate that specimens with closed spacing stirrup and steel fiber such as HC1 and HC2 have highest energy absorption compare with normal concrete beams (NN) even with normal beam with closed spacing stirrups (NC).

**Table 8:** Energy and ductility of the specimens.

Specimen	$\mu =\frac{{\Delta }_{\text{u}}}{{\Delta }_{\text{y}}}$	Increasing $\mu$ Compare to ${\mu }_{\left(N.N\right)}$ (%)	${E}_{y}$ (kN mm)	${E}_{u}$ (kN mm)	${\upmu }_{\text{E}}=\frac{{\text{E}}_{u}}{{\text{E}}_{\text{y}}}$	Increasing ${\mu }_{e}$ Compare to ${\mu }_{e\left(N.N\right)}$ (%)
NN	2	1	1715	3655	2.13	1
NC	2.12	6	1950	4405	2.25	6
HN1	3.67	83	3995	12,106	3.03	42
HC1	4.33	117	5522	21,827	3.95	85
HN2	3.4	70	3684	21,488	5.83	174
HC2	3.65	83	2992	20,273	6.77	218
2PHN1	3.3	65	4674	12,029	2.57	20
2PHC1	4.55	128	4111	13,500	3.28	54

Table 9 displays calculations, highlighting the significant improvement in energy ductility with increased fibers, emphasizing superior energy absorption capacity. Also, the ductility of 1% fiber beams with wide and closed spacing stirrups, subjected to two-point loading, 2PHN1, and 2PHC1, is lightly increased or decreased by less than 10% compared to HN1 and HC1 specimens.

**Table 9:** Loads details of the tested beams.

Group	Specimen notation	Experimental values		Bilinear method		Elastic stiffness k = P_y_/∆_y_ (kN/mm)		
		${P}_{y}$ (kN)	${\Delta }_{y}$ (mm)	${P}_{y}$ (kN)	${\Delta }_{y}$ (mm)	Exp	Bilinear	Exp/Bil
Group 1	NN	257	10.5	275	11.7	24.5	23.5	1.04
	NC	303	13.17	300	13	23	23.1	0.99
	HN1	347	23	455	32.0	15.1	14.2	1.04
	HC1	400	22.6	510	32.0	17.7	15.9	1.10
	HN2	370	24	510	34	15.4	15	1.03
	HC2	450	26	505	35.5	17.3	14.3	1.04
Group 2	2PHN1	405	23.5	515	31	17.2	16.6	1.04
	2PHC1	500	26	550	30	19.2	18.3	1.05

- **Stirrup spacing effect**

Reducing the stirrup spacing from d/2 to d/4 proved highly effective in enhancing both displacement and energy ductility. The reduction from d/2 in the NN specimen to d/4 in the NC specimen resulted in a remarkable 6% increase in both displacement and energy ductility. Simultaneously, the maximum load capacity rose by up to 14%. Additionally, reducing stirrup spacing in the 1% fiber volume specimens led to a substantial 34% increase in displacement and a 43% increase in energy ductility, coupled with a 20% boost in maximum load. In the case of closed-spacing specimens with 2% fiber (HC2), both displacement and energy ductility saw significant improvements, increasing by 13% and 44% respectively, when compared to their counterparts with wide-spacing stirrups (HN2). Moreover, the specimen 2PHC1, subjected to two-point loading, demonstrated noteworthy advancements. It displayed a substantial 14% increase in maximum load and exhibited impressive increases of 63% and 34% in displacement and energy ductility when compared to the wide-spacing stirrup beams 2PHN1. These results underscore the substantial benefits of reducing stirrup spacing in enhancing the structural performance and ductility of the tested specimens.

- **Steel fiber effects**

Introducing 1% fibers to normal concrete with wide-spaced stirrups (HN1) result in a remarkable 83% increase in displacement and a 42% increase in energy ductility compared to regular concrete (NN). Similarly, in specimen HN2, this modification leads to a substantial 70% increase in displacement and a remarkable 174% increase in energy ductility. However, increasing the fiber percentage from 1 to 2% in high-performance fiber-reinforced cementitious composite (HPFRCC) concrete caused a decrease in displacement ductility while improving energy ductility. Specifically, increasing the fiber content to 2% in wide-spaced stirrups (HN2) led to a 13% decrease in displacement ductility and a 130% increase in energy ductility compared to the specimen with 1% fiber (HN1). This trend is also observed in closed-spacing stirrup specimens; increasing the fiber content to 2% in HC2 led to a 34% decrease in displacement ductility and a 133% increase in energy ductility compared to the specimen with 1% fiber (HC1).

### Load-strain curve

**Fig. 16:**
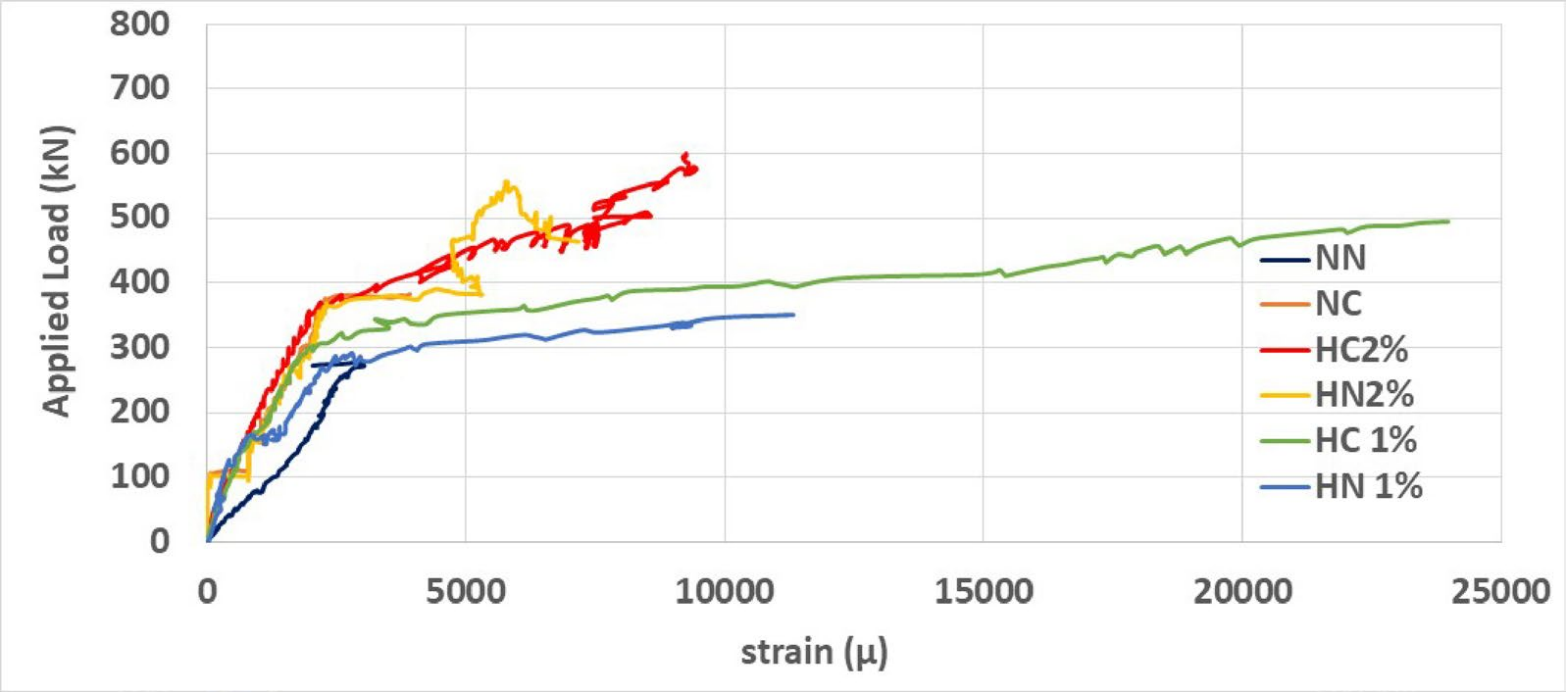
Load-strain of steel bars.

Figure 16 illustrates the maximum measured strain in bars at the critical section concerning the load for specimens. The graph depicts a notable slope change of approximately 2500 μm within the tensile region, indicating bars yielding a consistent result with the conducted bar tensile tests. High-performance beams exhibit significantly greater maximum tensile strains, surpassing those in normal concrete beams by approximately six times or more. Moreover, an increase in fiber and a reduction in stirrup spacing within the plastic region result in higher load capacities. Among the beams, the NN beam shows the lowest load at this strain, while the HC1 beam demonstrates the highest load.

### Elastic stiffness

**Fig. 17:**
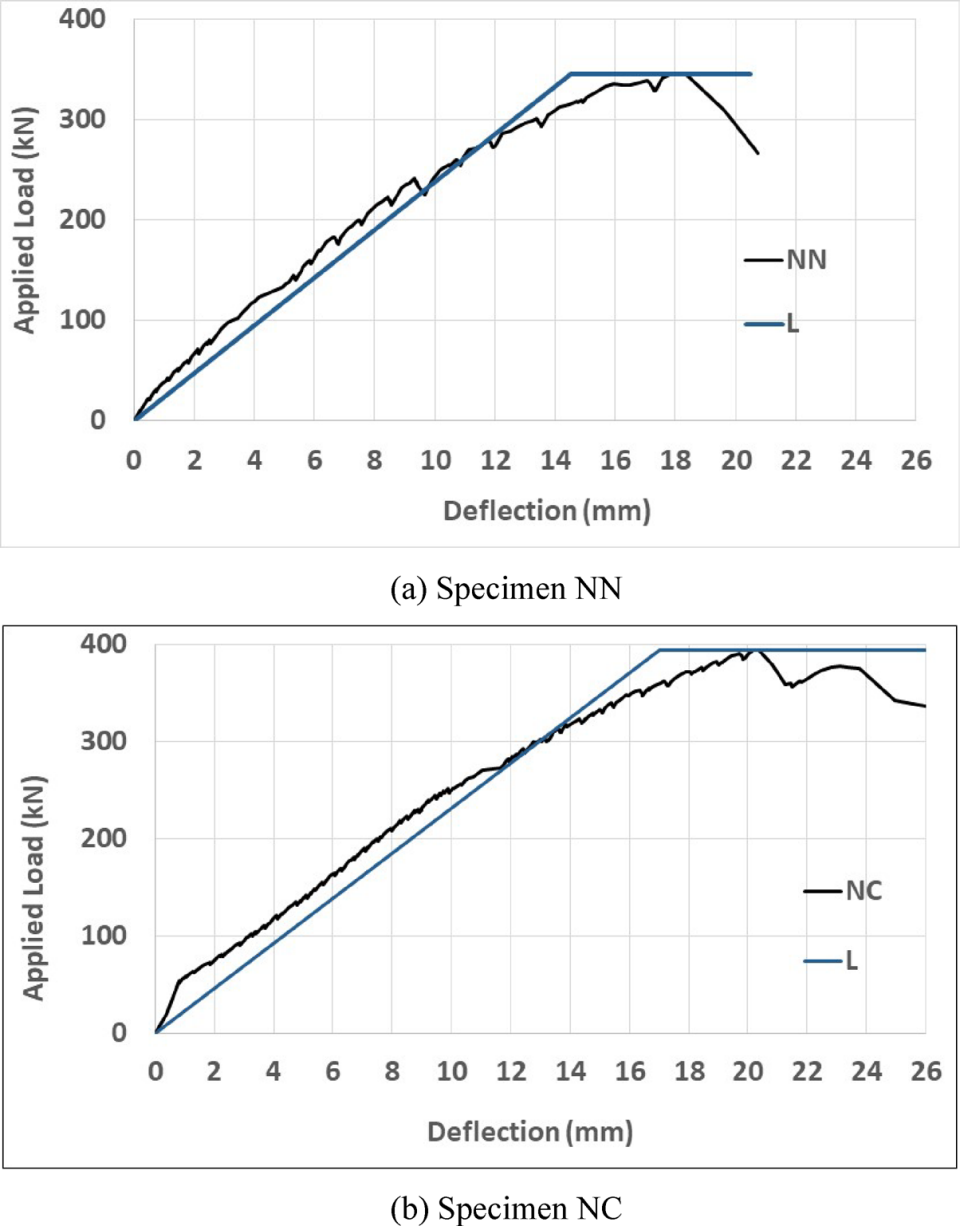
The bilinear load–displacement curves of non-fiber specimens.

The load–displacement diagrams of all specimens were plotted using the bilinear equivalent energy method, and an example of these curves for fiber-free beams is shown in Fig. 17. The applied yielding load (P_y_) and the corresponding displacement (∆_y_) were obtained for all specimens using this method. According to Park’s Bilinear Method, the correct position of the initial sloped (elastic) line is determined when the combined area of the two upper regions equals the area of the lower region. If the areas do not balance, the slope of the initial line must be iteratively adjusted through trial and error until it intersects the actual curve at the accurate yield point, defined by the yield displacement Δ_y_ and yield load P_y_.

The initial elastic stiffness (k = P_y_/∆_y_) was calculated using the bilinear diagram, and the experimental results were compared with those values obtained here; the results are presented in Table 9. The maximum difference between experimental and bilinear stiffness is 10%. And also, confined specimens had 10% more stiffness than that of normal and non-confined specimens. The stiffness of fiber-reinforced concrete specimens shows an average of 55% less stiffness than that of specimens with normal concrete. The 2PHN1 and 2PHC1 specimens subjected to two-point loading show up to a 20% increase in elastic stiffness compared to companion beams subjected to middle-span loading.

### Curvature ductility and effective flexural stiffness

**Fig. 18:**
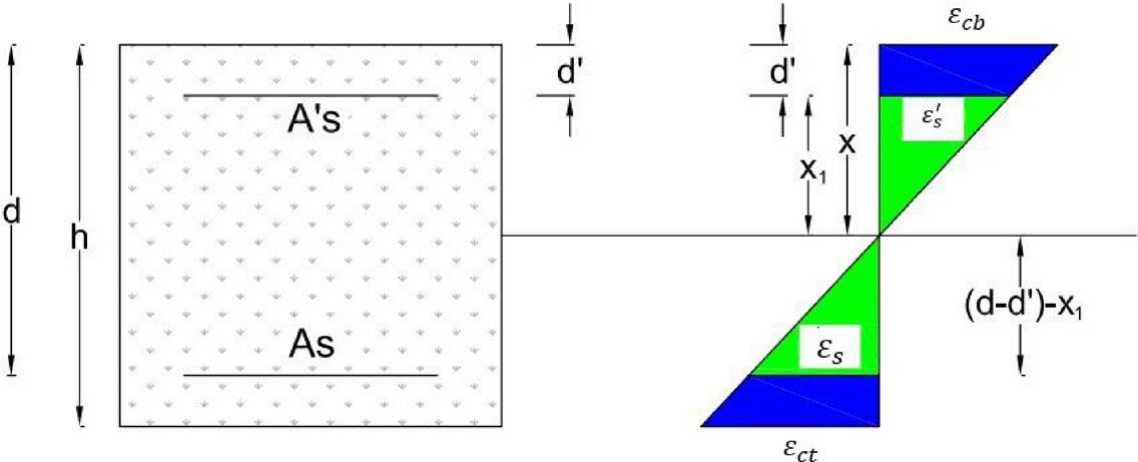
Changes in the strain at different heights in the middle of the section.

By reading the strain gauges at two levels, tension and compression, the strain variation of the bars is plotted during various loading stages, as shown in Fig. 18. The total height of the beam (h) is 25 cm, and the effective height (d) is 21 cm, with a cover of d´ equal to 3 cm; therefore, (d–d´) is equal to 19 cm. The concrete strain values are calculated using related equations. Curvature is defined as the ratio of the compressive strain of concrete to the depth of the compressive zone of concrete, $x$, for each case of applied load and is denoted as φ = ${\varepsilon }_{c}$/$x$. Curvature ductility is defined as the ratio of ultimate curvature φ_u_ (at ultimate load) to the yielding curvature φ_y_ (at yielding load) and is denoted as φ_u_/φ_y_ = μ. The moment values are calculated using the equation $M=\frac{P}{8}\times L=0.225P$. By plotting the moment–curvature curve and its bilinearization, Effective flexural stiffness is defined as M/φ = (EI)_ef_.

Figure 17 represents the changes in strain at different heights as measured by the strain gauges. Strain variations along the height of the beam during different loading conditions, including yielding loading P_y_, maximum loading P_max_, and ultimate load P_u_ at 0.8 times the maximum load, have been obtained. Based on this information, curvature values and related ductility are determined separately for each loading stage of each sample. Concrete compressive strains in various stages of steel yielding, maximum load, and ultimate load (concrete crushing) are respectively equal to ${\varepsilon }_{cy}$, ${\varepsilon }_{co}$, and ${\varepsilon }_{cu}$, so all of which are presented in Table 10. By reducing stirrup spacing in the plastic length (l_o_) and increasing the percentage of fibers, displacement, energy, and curvature ductility were increased. In HC2 and 2PHC1, it was observed a 112% and 100% increase in ductility. In fiber-reinforced beams, the final displacement significantly increases, leading to enhanced ductility. Moreover, the curvature ductility of HC2 2PHC1 beams was increased to 108 and 80%. In fiber-reinforced beams, the final curvature significantly increases, leading to enhanced deformability. In fiber-reinforced beams, the final energy increases significantly, resulting in enhanced energy ductility.

**Table 10:** Amount of curvature ductility of all specimens.

Specimen notation	${\varepsilon }_{cy}$ (μm)	${x}_{y}$ (mm)	${\varphi }_{y}$ (1/mm) *10^–5^	${\varepsilon }_{c0}$ (μm)	${\varepsilon }_{cu}$ (μm)	${x}_{u}$ (mm)	${\varphi }_{u}$ (1/mm) *10^–5^	${\upmu }_{ \varphi }$	Increasing ${\upmu }_{\varphi }$ compare to ${\upmu }_{\varphi NN}$ (%)
NN	2012	75	26.83	3684	5400	103	49.33	1.85	-
NC	2338	78	29.97	4619	6063	109.5	57.27	1.94	5
HN1	3475	94	36.97	5954	7145	85.4	83.67	2.26	22
HC1	3654	92.8	39.38	5998	8646	91.8	94.18	2.39	30
HN2	3800	90	42.22	6998	9917	98	101.19	2.4	30
HC2	4008	88.4	45.35	5910	10,466	92.3	113.4	2.5	35
2PHN1	3675	90	40.80	5994	7345	84.4	87	2.13	15
2PHC1	3754	88.8	42.27	6245	8746	92.7	94.35	2.23	21

In this section, using strain gauges installed on the tension bars at a distance of 50 mm from the support and the tension bar at the midpoint, the moment–curvature curves were plotted. To find the effective flexural stiffness, a bilinear approach on each moment–curvature curve is adopted. The corresponding values are presented in Table 11, calculated based on Eq. 1^25,35^. Finally, the obtained coefficients are compared with the ACI-318-19 and ACI 363 coefficients.

1 $$\begin{gathered} E_{NN} = 4700\sqrt {f_{c}^{\prime} } \quad E_{FRC} = \left( {3320\sqrt {f_{c}^{\prime} } + 6900} \right)\left( {\frac{{w_{c} }}{2300}} \right)^{1.5} \hfill \\ I_{g} = \frac{{bh^{3} }}{12}\quad EI_{eff} = \frac{{M^{\prime}_{y} }}{{\varphi^{\prime}_{y} }}\quad \alpha = \frac{{EI_{{_{eff} }} }}{{EI_{g} }} \hfill \\ \end{gathered}$$

**Table 11:** Effective flexural stiffness of tested beams.

Group	Specimen notation	At the support				At the middle			
		${{M}^{\prime}}_{y}$ (kN m)	${{\varphi }^{\prime}}_{y} \left(\frac{1}{\text{mm}}\right).{10}^{-6}$	${{E{I}_{eff}=M}^{\prime}}_{y}$/${{\varphi }^{\prime}}_{y}$ $\left(\text{N}.{\text{mm}}^{2}\right)$ 10^13^	$\alpha =\frac{E{I}_{eff}}{E{I}_{g}}$	${{M}^{\prime}}_{y}$ (kN m)	${{\varphi }^{\prime}}_{y} \left(\frac{1}{\text{mm}}\right).{10}^{-6}$	${{E{I}_{eff}=M}^{\prime}}_{y}$/${{\varphi }^{\prime}}_{y}$ $(\text{N}.{\text{mm}}^{2})$ × 10^13^	$\alpha =\frac{E{I}_{eff}}{E{I}_{g}}$
Group1	NN	68.36	18.25	0.375	0.29	66.19	21.42	0.31	0.24
	NC	64.42	20	0.322	0.322	76.21	23.73	0.32	0.25
	HN1	67.56	10.4	0.65	0.5	74.35	14.38	0.52	0.4
	HC1	77.74	11.8	0.66	0.51	72.41	22.75	0.32	0.25
	HN2	83.35	16.63	0.51	0.39	55.35	13.22	0.42	0.33
	HC2	89.44	17.37	0.52	0.4	91.3	16.7	0.55	0.43
Group2	2PHN1	83	17	0.49	0.38	60	12	0.5	0.39
	2PHC1	130	25	0.52	0.4	65	12	0.54	0.42

In all beams, by bilinearizing the moment–curvature curves near the support (50 mm from the support), at a distance of 150 mm and the midpoint of the beam, the yield values for both moment (${{M}^{\prime}}_{y}$) and curvature (${{\varphi }^{\prime}}_{y}$) are obtained. Then, the yield moment to yield curvature ratio is calculated, which represents the effective flexural stiffness. Ultimately, the ratio of effective flexural stiffness to total stiffness (defined as $\boldsymbol{\alpha }$) is calculated and compared with the values recommended by the ACI 318–19 code (minimum equal to 0.35), all of which are presented in Table 12. The ratio of effective flexural stiffness to total stiffness (defined as $\boldsymbol{\alpha }$) at the support and midpoint of normal concrete beams with wide spacing between the stirrups (NN) was determined to be 0.27 and 0.23, respectively, through bilinearization of the moment–curvature curves. These values are smaller than the recommended values in the ACI code. Therefore, special attention is needed for these normal concrete beams with wide spacing between stirrups.

**Table 12:** Plastic hinge length of specimens.

Specimen	NN	NC	HN1	HC1	HN2	HC2	2PHN1	2PHC1
Plastic hinge length (mm)	202.18	212.11	236.49	253.06	272.68	296.6	208.11	222.7
$\frac{lp}{{lp}_{NN}}$	1	1.05	1.22	1.35	1.35	1.47	1.03	1.1

These parameters in normal concrete beams with closed spacing between stirrups (NC) were slightly lower than the corresponding values in NN beams, mainly due to the higher number of stirrups at the supports. Adding fibers will increase the effective stiffness of the beams, exceeding the minimum recommended value in the code. For instance, the values $\boldsymbol{\alpha }$ for HN1 and HC1 beams at the support were approximately 0.50, and at the midpoint, they were about 0.40, indicating more deformation With an increase in fiber percentage in HN2 and HC2 samples, although the values $\boldsymbol{\alpha }$ (approximately 0.41) were higher than those in normal concrete beams and even exceeded the recommended values in the code, they were lower than the corresponding values in beams with lower fiber percentages, indicating that more increasing fibers leads to a decrease in effective stiffness. Additionally, beams with 1% fibers, even though subjected to two-point loading, exhibited different behavior compared to similar beams subjected to one-point loading. The behavior of these beams was closer to beams with 2% fibers under one-point loading.

### Plastic hinge

**Fig. 19:**
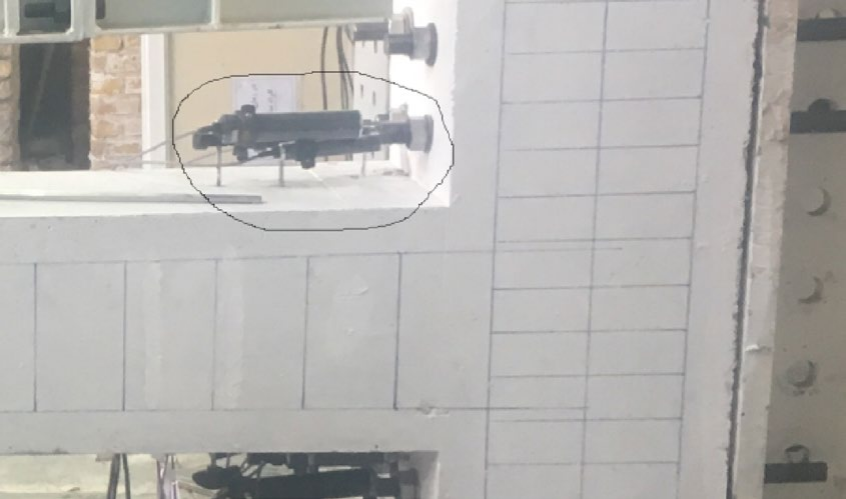
Threaded rods location on the beam.

**Fig. 20:**
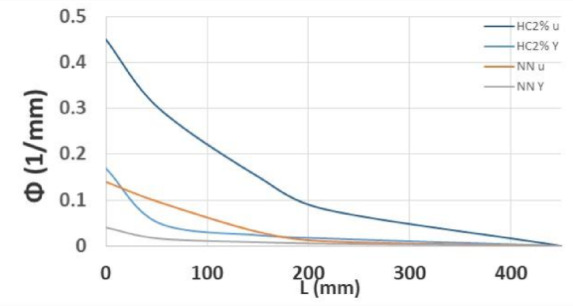
The curvatures of two specimens.

It’s crucial to recognize that sections in reinforced concrete are inherently non-homogeneous, a key distinction from homogeneous steel structures. Unlike steel sections, reinforced concrete sections exhibit varied behavior due to the performance of the embedded reinforcement. When a section’s capacity at a specific point along the RC beam is surpassed, the embedded bar yields. These points then act as hinges, transferring excess moments to sections under lower stresses as they rotate further. The process of moment redistribution is initiated by the formation of plastic hinges, where excess moments are transferred within a concrete member. This redistribution continues until the tensile bars at a specific point along the bending member reach their yield threshold, marking the transition into a mechanism and indicating a state close to instability. To measure beam rotation and the plastic hinge, specific bars and LVDTs were positioned at intervals from the support column, as shown in Fig. 19. The curvatures at different stages of loads were calculated based on the obtained data. Using this information, the curvature-length relationship was established, enabling the calculation of the plastic hinge length through the formula ${l}_{p}=\frac{{\theta }_{p}}{{\varnothing }_{u}-{\varnothing }_{y}}=\frac{{\theta }_{u}-{\theta }_{y}}{{\varnothing }_{u}-{\varnothing }_{y}}$. Results indicate that incorporating fibers into normal concrete, creating high-performance concrete, can increase the plastic hinge length by up to 46%, as shown in Fig. 20. Additionally, reducing stirrup spacing within the plastic region leads to a further increase in this length, as detailed in Table 12.

The results of Table 8 show that reducing the stirrup spacing increased ductility by only 6%, whereas adding just 1% and 2% steel fibers to beams with large stirrup spacing increased ductility on average by 60% and 120%, respectively. Moreover, the combined effect of reducing stirrup spacing and adding 1% and 2% steel fibers led to average increases of 100% and 150% in displacement and energy ductility, respectively. These increases in ductility are clearly observable in the cracking patterns and the higher number of micro-cracks shown in Figs. 7, 8, 9, 10, 11, 12, 13 and 14, where greater ductility resulted in a softer failure mode and an increased plastic hinge length.

**Fig. 21:**
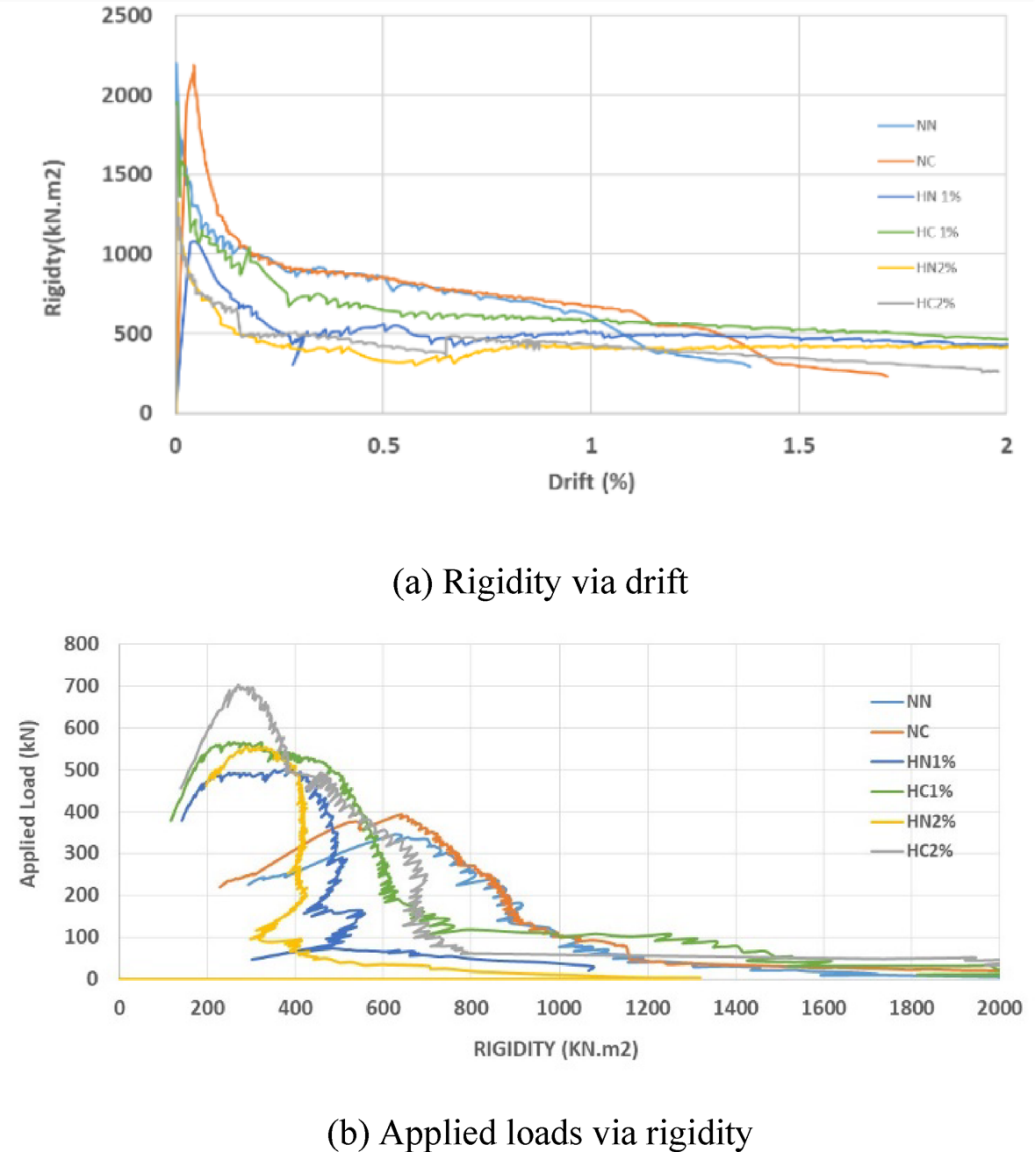
Rigidity, drift, and applied loads of tested beams.

To calculate the actual rigidity, the real displacements at the center of the beams were utilized, employing the formula ($P{L}^{3}/(192\Delta )$. Subsequently, the corresponding rigidity-drift and load-rigidity curves were plotted for all the beams. It was observed that in normal concrete beams, the rigidity is higher at similar loads. However, after the initiation of cracking, there is a significantly more pronounced reduction in rigidity (P/Δ). Conversely, in high-performance concrete beams, once the rigidity reaches 0.2 of its initial value, the reduction in rigidity becomes negligible, persisting until the completion of the loading cycle and eventual failure. Consequently, as depicted in Fig. 21, the effective rigidity in normal concrete beams with fixed ends can be considered as 30% of the initial rigidity, while in high-performance beams, it is approximately 20% of the initial rigidity, yet more stable, as illustrated in Fig. 20a and b. Drift Percentage is defined as 100∆/L, and Fig. 20b indicates that the total rigidity of specimens was decreased with the addition of fibers at a specified drift. Furthermore, it is noteworthy that increasing the spacing has a more pronounced effect on reducing rigidity.

### Toughness index

The parameters of the Toughness index of all tested beams are calculated using ASTM C1018^36^. The first crack displacement value (Δ_cr_) is obtained from Table 8, and the area under the load–displacement curve up to this point (U_0_) is determined. Therefore, the areas under the load–displacement curves up to 3 times the first crack displacement (U_5_), 5.5 times the first crack displacement (U_10_), and 15.5 times the first crack displacement (U_30_) are calculated. By dividing the areas of U_5_, U_10_, and U_30_ by the area up to the first crack displacement (U_0_), the indexes I_5_, I_10_, and I_20_ are calculated, respectively, and the corresponding values are provided in Table 13. The first crack resistance characterizes the behavior of fiber-reinforced concrete up to the initiation of cracks in the concrete, while the Toughness index indicates the concrete’s strength after the first crack until a specified displacement. Residual strength factors, directly derived from the Toughness index according to Eqs. (3) and (4), represent the level of remaining strength after the initial crack within the specified displacement range and are expressed as a percentage of the cracking load.

**Table 13:** Toughness index of all tested beams.

Group	Specimen notation	${I}_{5} (\text{kN}.\text{mm})$	${I}_{10} (\text{kN}.\text{mm})$	${I}_{30} (\text{kN}.\text{mm})$	$\frac{{I}_{10}}{{I}_{5}}$	$\frac{{I}_{30}}{{I}_{10}}$	${R}_{\text{5,10}}$
Group 1	NN	6.66	22.7	129.8	2.52	5.72	320.9
	NC	6.11	16.46	101.8	2.57	5.76	206.9
	HN1	7.19	23.42	116	3.26	4.95	324.6
	HC1	7.85	25.19	143.8	3.25	5.71	346.2
	HN2	8.5	29.82	199	3.51	6.67	426.7
	HC2	9.5	32.84	230.2	3.46	7.01	466.8
Group 2	2PHN1	6.69	20.42	97	3.05	4.75	274.6
	2PHC1	6.85	21.19	102.8	3.1	4.85	286.8

The Toughness and crack resistance depend on factors such as the type and quantity of fibers. In determining the most suitable Toughness index as a performance measurement criterion for a specific use, the service level required concerning cracking and displacement must be considered. An index proportional to the service conditions should be selected. The values of the Toughness index and crack resistance are used for comparing the performance of different fiber-reinforced concretes during the proportioning process or research and development. They are also employed for quality control of the concrete in use to confirm compliance with structural specifications or for evaluating concrete quality in service. These indices can also be used to determine the percentage of elastoplastic behavior of the specimens.

In a fully elastoplastic behavior scenario, I_5_ equals 5, I_10_ equals 10, and I_30_ equals 30, and the residual factor in this case is 100, which can be calculated from the relationship. The concept of the Toughness index expresses the energy absorption capacity of the beams within the specified displacement range and is directly related to the geometric conditions and loading of the beam. The residual strength factors represent the average load after cracking and are expressed as a percentage of the initial crack load within the specified displacement range, which can be useful for evaluating concrete quality, assessing mix design quality, and comparing two similar concretes.

2 $$R_{5,10} = 20\left( {I_{10} - I_{5} } \right)$$

3 $$R_{30,10} = 10\left( {I_{30} - I_{10} } \right)$$

The results indicate that the values of the indices I_5_ and I_10_ are higher than the standard values of I_5_ and I_10_ given in ASTM C1018. This suggests that up to 5.5 times the crack displacement, the crack resistance performance of these specimens is better than elastoplastic behavior, and the load capacity increases within this range. Furthermore, the residual strength factor R_5,10_ for these beams is higher than 100, confirming the aforementioned statement. The Table results show an increase in all indices compared to the reference beam, indicating an increase in energy index with an increase in fiber percentage and a decrease in stirrup spacing at these distances. The ratio of I_10_/I_5_ for the tested beams is in the range of 52.2 to 46.3, indicating that the behavioral pattern of these specimens approaches fully plastic behavior conditions. In the two beams loaded at two points, the residual strength parameter is lower than the other beams. This is due to the change in loading and energy parameter values of these two beams, which will undoubtedly be higher compared to a regular concrete beam under the same loading conditions.

### Nominal moment capacity versus experimental amounts

**Fig. 22:**
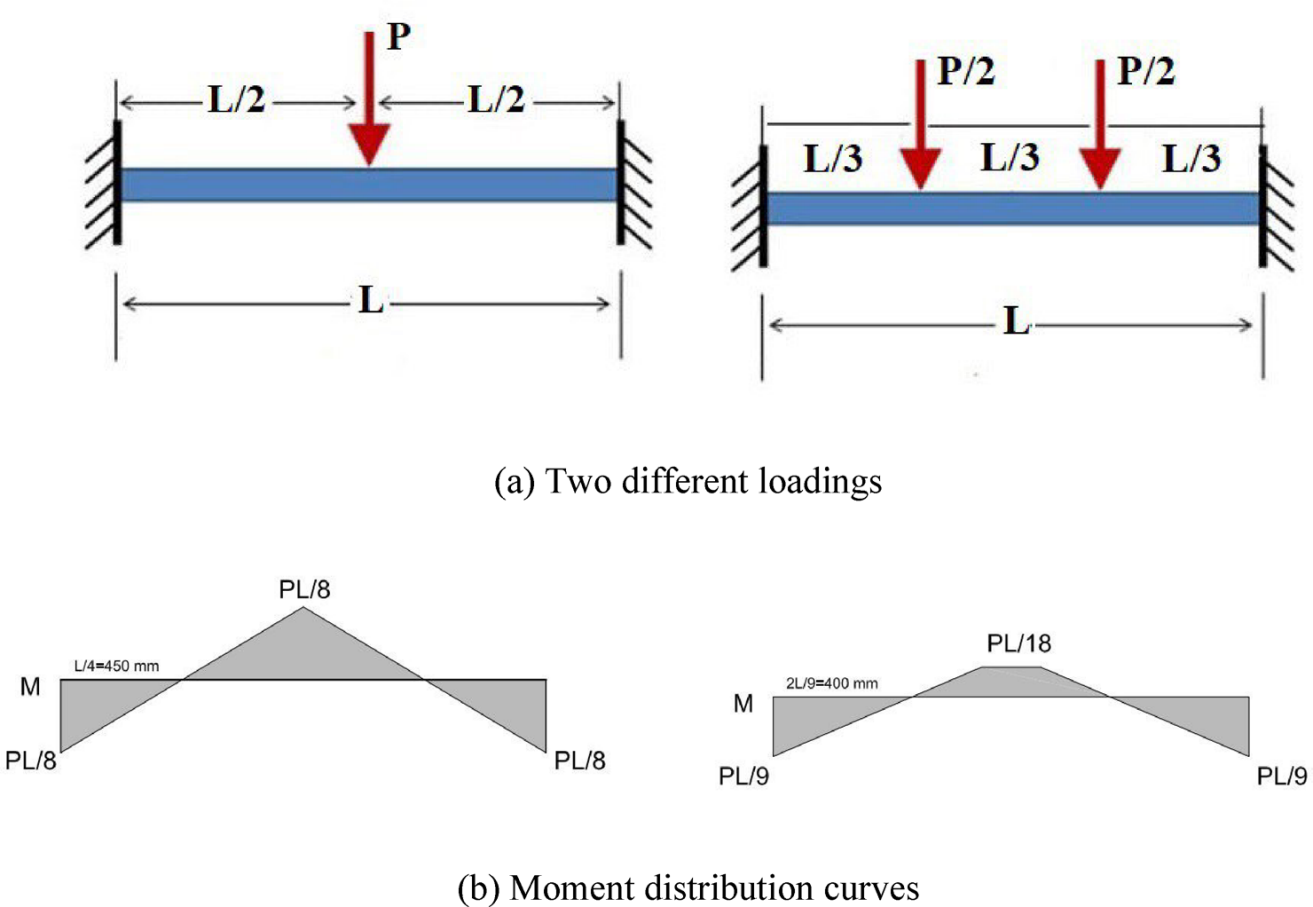
Different loading and related moment curves.

The experimental beams in two different groups, similar to Fig. 22, were loaded with concentrated loads P applied either at the midspan of the beams or with two concentrated loads P/2 at one-third spans. Maximum experimental moments were obtained based on the maximum loads and relevant moment formulas (PL/8 or PL/9). The beams had both tensile and compressive reinforcements, and the tensile steel would yield before the concrete was crushed.

**Fig. 23:**
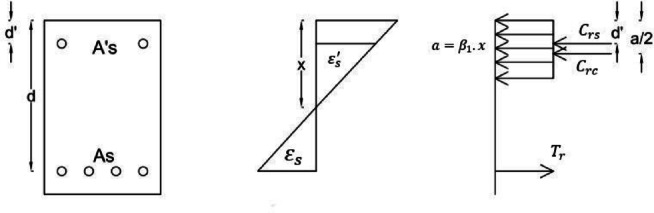
Internal strain, stress, and loads of beams.

The compressive steel might not reach its yield strength. In the support region, fewer bars were used in the tension zone (top of the beam). However, in the tension zone of the middle of the beam, more bars were used to strengthen the middle of the beam, initiating the final mechanism and forming the plastic hinge. The simultaneous occurrence of 3 mechanisms at the two supports and the middle of the beam prevented to examination of the redistribution of moments from the supports to the middle of the beam. This is the reason for the positive and negative differences in the moment capacities of the beams. Equation (4) from the ACI318-19 code and Fig. 23 were used to calculate the nominal flexural moments in ordinary concrete beams without fibers. The nominal flexural moment of fiber-reinforced beams was calculated using two equations from ACI 544 and the studies by Khalil and Tyfoor^37^.

4 $$\begin{gathered} a = \frac{{(f_{y} A_{s} - A_{s}^{\prime} \left( {f_{y} - 0.85f_{c}^{\prime} } \right)}}{{0.85f_{c}^{\prime} b}} \hfill \\ c = \frac{a}{{\beta_{1} }} < \frac{600d}{{600 + f_{y} }}\quad \left( {\beta_{1} = 0.69} \right) \hfill \\ mover\frac{{d^{\prime}}}{a} > \frac{1}{{\beta_{1} }}\left( {1 - \frac{{f_{y} }}{600}} \right) \hfill \\ C_{s} = A_{sf}^{\prime} *\left( {f_{s}^{\prime} - 0.85f_{c}^{\prime} } \right) \hfill \\ C_{c} = 0.85f_{c}^{\prime} ba \hfill \\ M_{n} = A_{sf}^{\prime} *\left( {f_{s}^{\prime} - 0.85f_{c}^{\prime} } \right)\left( {d - d^{\prime}} \right) + 0.85f_{c}^{\prime} ba\left( {d - \frac{a}{2}} \right) \hfill \\ \end{gathered}$$

**Fig. 24:**
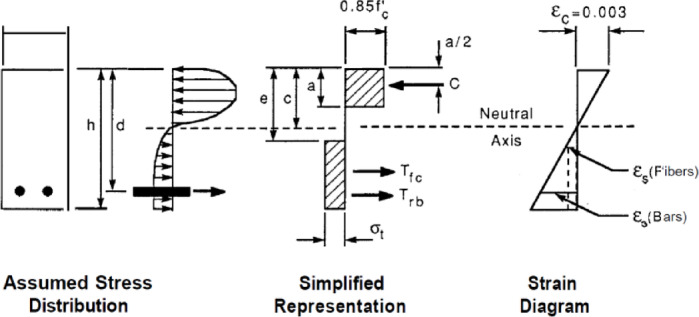
Design assumptions for the analysis of FRC concrete beams based on ACI 544R.

Formula (5) was used to calculate the nominal flexural moments of RC beams with bars and fibers, according to the provisions of the ACI 544 code^38^. The main assumptions of the design are illustrated in Fig. 24.

5 $$\begin{gathered} M_{n} = A_{s} f_{y} \left( {d - \frac{a}{2}} \right) + \sigma_{t} b\left( {h - e} \right)\left( {\frac{h}{2} + \frac{e}{2} - \frac{a}{2}} \right) \hfill \\ e = (\varepsilon_{s} \left( {fibers} \right) + 0.003){\raise0.7ex\hbox{$c$} \!\mathord{\left/ {\vphantom {c {0.003}}}\right.\kern-0pt} \!\lower0.7ex\hbox{${0.003}$}} \hfill \\ \sigma_{t} = 0.00772{\raise0.7ex\hbox{$l$} \!\mathord{\left/ {\vphantom {l {d_{f} }}}\right.\kern-0pt} \!\lower0.7ex\hbox{${d_{f} }$}}\rho_{f} F_{be} \;{\text{MPa}} \hfill \\ \end{gathered}$$

**Fig. 25:**
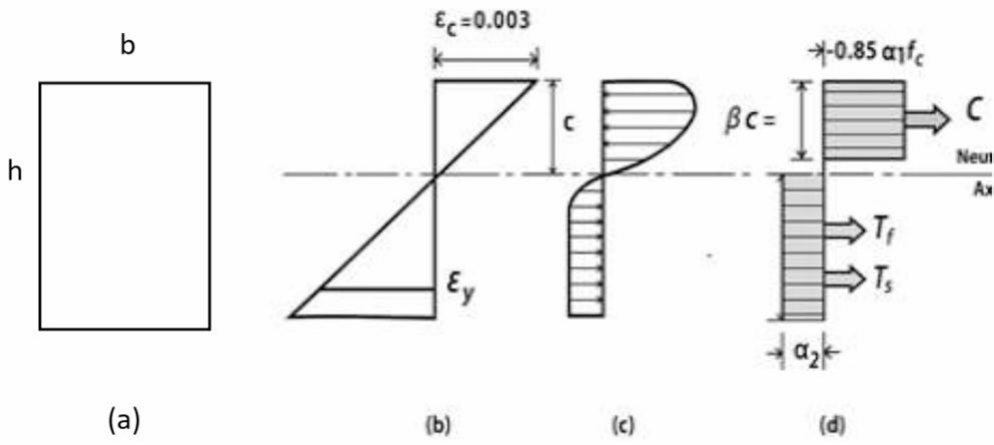
Design assumptions for the analysis of FRC Concrete beams based on the Khalil and Tyfur method.

The following parameters are defined for these equations. *l*, *d*_*f*_, ${\rho }_{f}$, *F*_*be*_ are length, diameter, percentage, and bonding Fiber. *a* is equivalent rectangular stress block. c is the distance from the Compressive strain to the neutral line. b, h and d are beam width, total height, and effective height. e is the distance from the Compressive strain to the top of a tensile rectangular block of fibers. ${\varepsilon }_{s}, {A}_{s}$, *E*_*s*_, and *T*_*rb*_ are the tensile strain, steel bar area, elastic modulus of steel bar, and tensile force of steel bars. ${\epsilon }_{c}, {f}_{c}^{\prime}$, and C are compressive strain, the strength of concrete, and compressive force. ${\sigma }_{t}$ and *T*_*fc*_ are the tensile stress and force of the fiber block. In the method proposed by Khalil and Tyfoor, Eqs. 6 were utilized to calculate the nominal moment of RC beams with steel bars and fibers. The main design assumptions are illustrated in Fig. 25.

6 $$\begin{gathered} M_{N} = A_{s} f_{y} \left( {d - c} \right) + \sigma_{t} \left( {h - c} \right)b\left( {\frac{h - c}{2}} \right) + \lambda f_{c}^{\prime} \beta_{1} cb{ }\left( {{\text{c}} - \frac{{\beta_{1} c}}{2}} \right) + A_{s}^{\prime} f_{y} \left( {c - d^{\prime}} \right) \hfill \\ c = \frac{{A_{s} f_{y} + \sigma_{t} bh}}{{\left( {\lambda \beta_{1} f_{c}^{\prime} b + \beta_{1} A_{s}^{\prime} f_{y} + \sigma_{t} b} \right)}} \hfill \\ \sigma_{t} = 2\eta_{o} \eta_{b} \eta_{l} V_{f} \tau_{f} \frac{{l_{f} }}{{d_{f} }} \hfill \\ \eta_{l} = 1 - \left( {\frac{{\tan h\left( {0.5\beta l_{f} } \right)}}{{0.5\beta l_{f} }}} \right) \hfill \\ \beta = \sqrt {\frac{{2\pi G_{m} }}{{E_{f} A_{f} ln\left( {\frac{S}{{r_{f} }}} \right)}}} \hfill \\ S = 25\sqrt {\frac{{l_{f} }}{{V_{f} d_{f} }}} \hfill \\ \tau_{f} = 0/66\sqrt {f_{c}^{\prime} } \hfill \\ \end{gathered}$$

where ${V}_{f}$, Fiber ratio (less than 1); ${\tau }_{f}$, Average interfacial bond shear strength between fiber and matrix (MPa). Typical range: 2–8 MPa depending on fiber surface and matrix properties; ${l}_{f}$, Fiber length (mm); ${d}_{f}$, Fiber diameter (mm); ${\eta }_{b}$, Fiber bonding strength factor, equal 1 for smooth fiber and 1.2 for hooked fiber; ${\sigma }_{t}$, Ultimate tensile strength of fiber concrete; ${\eta }_{o}$, Rotating factor of fiber, equal to 0.41 for uniform distributed; ${\eta }_{l}$, Fiber length factor; ${G}_{m}$, Shear modulus of concrete; ${E}_{f}$, elastic modulus of fiber; ${r}_{f}$, Fiber radius; $\lambda$, Compressive stress block parameter, equal 0.85 for compressive strength more than 55 MPa; ${\beta }_{1}$, Compressive stress block parameter equal 0.65 to 0.85 for compressive strength more than 30 MPa, minimum 0.65.

The results of theoretical and experimental nominal flexural moments of the specimens and their comparison are presented in Table 14. The 1% fiber beams with wide and closed spacing stirrups, subjected to two-point loading (called 2PHN1 and 2PHC1), showed around 34 and 31% increase in experimental moment capacity in Table 15 and up to 31% increase in elastic stiffness compared to companion beams subjected to middle span loading (HN1 and HC1). Also, the ductility of 2PHN1 and 2PHC1 specimens was lightly increased or decreased by less than 10% compared to HN1 and HC1 specimens. By comparing the columns in Table 14, it can be observed that in the case without fibers, all results are very close to each other, indicating that due to the consistent assumptions, all equations are natural. In the case of concrete with fibers, according to ACI 544 and the method proposed by Khalil and Tyfoor, higher results are obtained due to the consideration of fiber effects compared to ACI 318-19. However, considering the significant difference with experimental results, modifying fiber effect coefficients, such as the bond strength factor and fiber rotation factor, could be effective in approaching experimental results. In this study, the author suggests using a modification factor of 5 for the bond strength factor in hooked steel fibers with 10 and 20-mm lengths, which shows very good agreement with the experimental results of this research (104 kN·m for 1% fibers and 120 kN·m for 2% fibers).

**Table 14:** Comparison of theoretically and experimental moments in the middle of the beams.

Specimen notation	M_n, ACI318_ (kN.m)	M_n, ACI544_ (kN.m)	M_n, Khalil_ (kN.m)	M_n,Exp_ (kN.m)	$\frac{{M}_{n} Exp}{{M}_{n} ACI 544}$	$\frac{{M}_{n} Exp}{{M}_{n} Khalil}$
NN	89.1	89.1	89.1	79	0.88	0.88
NC	89.1	89.1	89.1	90	1.01	1.01
HN1	-	90	93	108	1.2	1.16
HC1	-	90	93	127	1.4	1.37
HN2	-	95	97	127	1.33	1.31
HC2	-	95	97	161	1.69	1.66
2PHN1		90	93	145	1.61	1.66
2PHC1		90	93	166.5	1.85	1.91

**Table 15:** Comparison of main results of all beams specimens.

Specimen notation	$\frac{{P}_{max}}{{P}_{max\left(N.N\right)}}$	$\frac{\mu }{{\mu }_{\left(N.N\right)}}$	$\frac{{\mu }_{e}}{{\mu }_{e\left(N.N\right)}}$	$\frac{{\mu }_{\varphi }}{{\mu }_{\varphi NN}}$	$\frac{lp}{{lp}_{NN}}$	$\alpha =\frac{E{I}_{eff}}{E{I}_{g}}$	$\frac{{M}_{n} Exp}{{M}_{n} ACI 544}$
NN	1	1	1	1	1	0.24	0.88
NC	1.14	1.06	1.06	1.05	1.049	0.25	1.01
HN1	1.44	1.83	1.42	1.22	1.22	0.4	1.2
HC1	1.64	2.17	1.85	1.3	1.35	0.25	1.4
HN2	1.61	1.7	2.74	1.3	1.35	0.33	1.33
HC2	2.04	1.83	3.18	1.35	1.47	0.43	1.69
2PHN1	-	1.65	1.2	1.15	1.03	0.39	1.61
2PHC1	-	2.28	1.54	1.21	1.1	0.42	1.85

The reason for the higher experimental results is mainly due to the effects of plastic hinge and redistribution of moments, the compression effect on the increase of bearing capacity and moment, and the strain hardening of steel bars. Additionally, there is a better bond between concrete and fibers due to the smaller fibers used in research (10 mm) compared to the fibers used in the equations by other researchers, which were over 50 mm. When shorter fibers are used, the number of fibers in a specific volume of concrete increases, and finer cracks are formed, leading to higher tensile, flexural, and bond strengths of concrete^39^. The average experimental moments of HN1 and HC1 beams is 117 kN.m, approximately 24 kN.m more than the moment that is calculated based on the initial equation given by Khalil. So, to provide the new moment, the amount of ${\sigma }_{t}$ should be around 2.5 MPa. This can be adjusted by using supplementary factors such as increasing the bond strength factor or the fiber rotation factor, as shown in Eq. (7).

7 $$\sigma_{t} = \varphi \eta_{o} \eta_{b} \eta_{l} V_{f} \tau_{f} \frac{{l_{f} }}{{d_{f} }}\quad \eta_{b} = 1.8\quad \tau_{f} = 1.2\sqrt {f_{c}^{\prime} } \quad \varphi = 3\quad \eta_{o} = 0.5$$

**Fig. 26:**
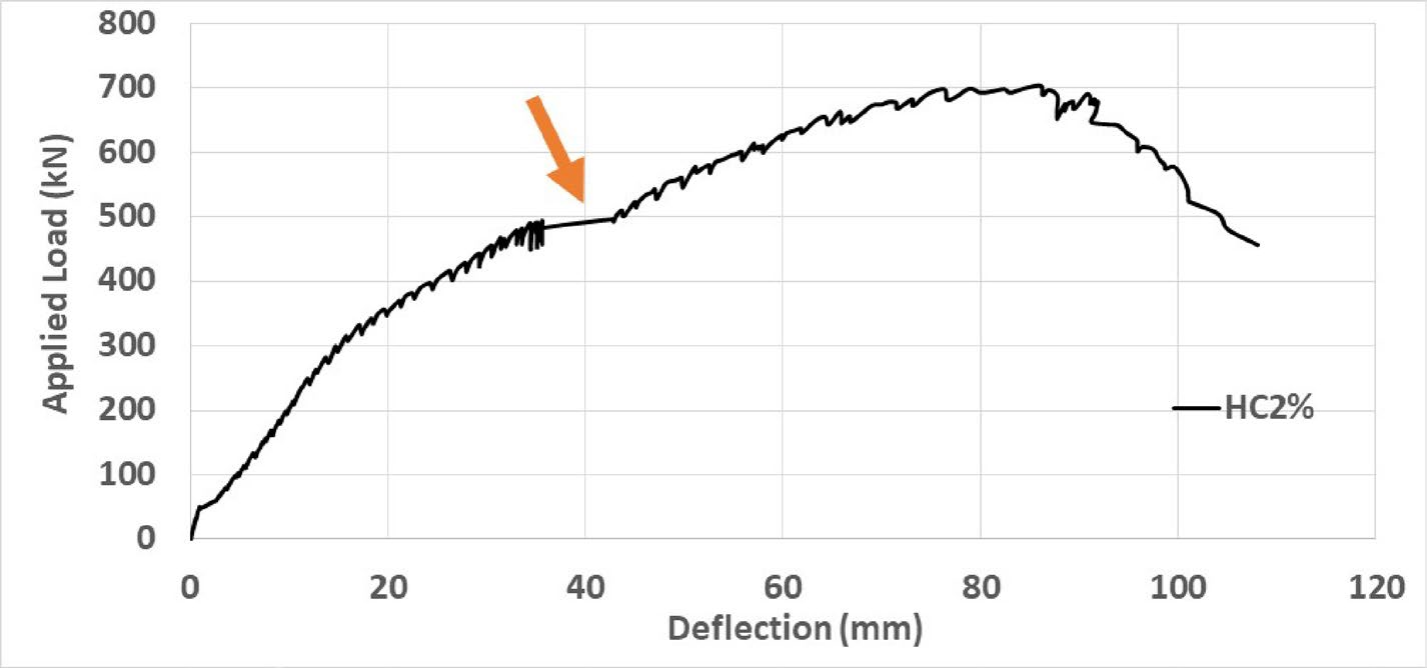
The phenomenon of strain hardening of fiber-reinforced concrete HC2.

To investigate the effect of strain hardening of high-strength concrete beams reinforced with steel fibers, Fig. 26 illustrates the load–displacement curve of beam HC2. In this graph, it can be observed that around a load of approximately 500 kN, the curve becomes horizontal and then increases in slope again. This phenomenon represents the strain hardening of fiber-reinforced concrete, and as the percentage of fibers increases, this effect becomes more significant and noticeable. In strain hardening behaviors, the rate of strain increase is always higher than the rate of load increase. This is crucial and effective in increasing ductility and also in preventing the brittle failure of concrete beams and issuing warnings before failure occurs.

#### General comparison of specimens

The results of all specimens are summarized in Table 15 and compared with the beam made of normal concrete with wide stirrup spacing (NN). The results show that reducing stirrup spacing slightly increases load capacity and other parameters. The substantial increases in all three measures of ductility—displacement, energy, and curvature up to 218% highlight the significant effect of steel fibers at both 1% and 2% dosages. For specimens subjected to concentrated loading, the plastic hinge length increased by up to 47% for beams with 2% fiber. While reducing stirrup spacing had no noticeable effect on stiffness, adding 1% and 2% fibers increased stiffness by up to 70%.

Buildings designed for seismic resistance require higher ductility and energy absorption to withstand greater deflection and drift. They also need longer plastic hinge lengths to accommodate more cracking before collapse. Experimental results on fixed-end beams show significant improvements when using HPFRCC concrete instead of normal concrete, even with wider stirrup spacing. Increasing stirrup spacing also facilitates easier concrete casting in practice. Therefore, the findings of this study are directly applicable to the seismic design of reinforced concrete frame beams, offering enhanced strength and ductility.

## Summary

In this study, the behavior of eight specimens, identical longitudinal reinforcement percentage, configuration, and similar compressive strength, was examined, encompassing two concrete types (normal and HPFRCC) and two variations of stirrup spacing and load configurations. The experimental investigation yielded the following results:

- Replacing normal concrete of the beam with compacted stirrup (spacing of stirrups equal to one-fourth of the effective height of the beam, d/4) in (NC) with 1% steel fiber HPFRCC in HC1 resulted in a 48, 40, 54, and 74% increase in yield and ultimate loads, displacement ductility, and energy absorption. When comparing companion specimens HN1 to NN with un-compacted stirrup spacing (d/2), it also led to a 45% and 44% increase in yield and ultimate loads.
- Increasing the volume fraction of steel fibers from 1 to 2% in beams with compacted stirrup (HC1 and HC2) resulted in a 12.5, 12.7, 7, and 126% increase in yield load, ultimate load, displacement ductility, and energy absorption, respectively. These increases for beams with uncompressed stirrup (HN1 and HN2) were 10, 11, 33, and 90%. The impact of increasing the fiber content on these ductility parameters is evident.
- In beams with uncompressed stirrups, the addition of 1% fibers resulted in a 35% increase in plastic hinge length, while 2% fibers increased it by 40%. This change in plastic hinge length shifted the fracture mode from low-ductility flexural behavior to complete flexural behavior in HPFRCC concrete beams. A novel theoretical relation for predicting the plastic hinge length in HPFRCC and conventional beams was developed. This relation is especially valuable considering the absence of such a relationship in the case of high-strength HPFRCC concrete beams and may be proposed to relevant standards institutes.
- Reducing the stirrup spacing from d/2 to d/4 in conventional concrete resulted in only a 5% increase in plastic hinge length. However, when reinforced fiber concrete was used, there was a substantial 47% increase in plastic hinge length observed at the beam ends in HC2, leading to moment redistribution.
- Reducing the spacing of stirrups from d/2 to d/4 of beams with 1% fiber reinforcement, subjected to a two-point load (2PHN1, 2PHC1) resulted in a 12, 47, 23, and 31% increase in yield load, ultimate load, displacement ductility, and energy absorption, respectively.
- Changing the load distribution from a single point to two points in a beam with 1% steel fibers resulted in up to 34, 31, and 10% increases in experimental moment capacity, elastic stiffness, and ductility.
- Reducing the stirrup spacing from d/2 to d/4 (the effective height) in conventional concrete led to a 50% increase in energy absorption; however, the inclusion of fibers resulted in a remarkable increase of nearly 13 times greater energy absorption compared to companion beams. This highlights the extraordinary impact of fibers on energy absorption. In beams subjected to a two-point load, the addition of fibers increased energy absorption by up to 3.7 times.

## Data Availability

The data are available from the first author, Mohammad Kazem Sharbatdar, upon reasonable request.
